# Mutational signature-based classification uncovers emerging oral cancer subtypes with distinct molecular patterns

**DOI:** 10.1038/s41368-026-00437-4

**Published:** 2026-04-24

**Authors:** Sophie Deneuve, Béatrice Fervers, Julia S. Bruno, Emma Bach, Sergey Senkin, Gabrielle Goldman-Lévy, Christine Carreira, Israa Laklouk, Rong Hu, Liacine Bouaoun, Olivia Pérol, Bérénice Chavanel, Lingeng Lu, Taja Lozar, Tarik Gheit, Paul F. Lambert, Isabelle Coste, Toufic Renno, Jiri Zavadil, François Virard

**Affiliations:** 1 Department of Otolaryngology-Head and Neck Surgery, CHU Rouen, Rouen, France; 2 QuantIF-LITIS Team EA4108, University of Rouen, Rouen, France; 3 Centre Léon Bérard, Département Prévention Cancer Environment, INSERM U1296 Unit Radiation: Defense, Health Environment, Lyon, France; 4 International Agency for Research on Cancer WHO, Epigenomics and Mechanisms Branch, Lyon, France; 5 Department of Maxillofacial and Facial Plastic Surgery, Lyon Sud Hospital, Hospices Civils de Lyon, Pierre-Bénite, France; 6 International Agency for Research on Cancer WHO, Evidence Synthesis and Classification Branch, Lyon, France; 7 Department of Pathology and Laboratory Medicine, David Geffen School of Medicine at University of California, Los Angeles, Los Angeles, CA USA; 8 Department of Pathology and Laboratory Medicine, University of Wisconsin School of Medicine and Public Health, Madison, WI USA; 9 International Agency for Research on Cancer WHO, Environment and Lifestyle Epidemiology Branch, Lyon, France; 10 Department of Chronic Disease Epidemiology, Yale School of Public Health, Medicine, New Haven, CT USA; 11 Center for Biomedical Data Science, Yale University, New Haven, CT USA; 12 Yale Cancer Center, Yale University, New Haven, CT USA; 13 McArdle Laboratory for Cancer Research, University of Wisconsin School of Medicine and Public Health, University of Wisconsin, Madison, WI USA; 14 University of Ljubljana, Ljubljana, Slovenia; 15 INSERM U1052–CNRS UMR5286, Cancer Research Center, University Claude Bernard Lyon 1, Centre Léon Bérard, Lyon, France; 16 Department of Oral Biology, Faculty of Dentistry, Lyon 1 University, Lyon, France; 17 Odontology Center, Hospices Civils de Lyon, Lyon, France

**Keywords:** Oral cancer, Cancer genomics, Genome informatics

## Abstract

Tobacco use, alcohol consumption, and infection with human papilloma virus (HPV) are well-established risk factors for head and neck squamous cell carcinomas (HNSCC). However, the incidence of oral cancer, particularly in the mobile tongue, has been rising in non-smoker/non-drinker and HPV-negative patients, suggesting the emergence of a new clinical entity. To understand in molecular terms this subtype of oral cavity squamous cell carcinomas (OCSCC) with no-identified risk factor (NIRF), we analyzed the available public head and neck cancer multi-omics data. We identified mutational signatures that stratified 253 OCSCC and 94 laryngeal cancer cases, used as tobacco-only-related controls, according to their clinico-pathological characteristics. We show that tobacco, depending on the anatomical site, triggers distinct mutational processes and further demonstrate that the single-base-substitution (SBS) signature SBS16 in OCSCC is associated with tobacco smoking, reflecting the combined effects of smoking and drinking. Importantly, we identified a tongue cancer-enriched NIRF OCSCC subgroup exhibiting significantly increased endogenous clock-like mutagenesis, while another NIRF subgroup manifested with elevated apolipoprotein B mRNA editing enzyme catalytic polypeptide-like (APOBEC)-associated mutagenesis. Both NIRF OCSCC subgroups harbored specific cancer driver mutations and distinct methylation patterns, which differed from those observed in OCSCC linked to traditional HNSCC risk factors, reflecting unique molecular programs underlying disease development. Specifically, NIRF-OSCC exhibited pronounced immune evasion strategies and antimicrobial transcriptomic responses. Our study presents the first molecular and genomic characterization of the emerging NIRF OCSCC subtype likely driven by increased endogenous mutagenesis and responses to microbial insults. These findings warrant future detailed investigations into etiology and have implications for clinical management and cancer prevention.

## Introduction

Oral cancer incidence rates for both sexes have shown a significant increase from the mid-2000s until the latest (2015–2019) National Cancer Institute survey.^1^ Originating from the oral mucosa, most of these malignancies are squamous cell carcinomas (SCC), a subgroup of the head and neck squamous cell carcinomas (HNSCC). Tobacco use and alcohol consumption, as well as infection with human papilloma virus (HPV) are the major risk factors for HNSCC.^2^ The particular clinical and histological presentations and better prognosis of HPV-positive cancers of the oropharynx have prompted the consideration of two distinct HNSCC subtypes: a HPV-positive HNSCC, mainly at the oropharyngeal subsite, and the conventional group of HPV-negative HNSCC at different anatomical sites associated with exposure to tobacco-derived carcinogens, excessive alcohol consumption, or both.^3^ A global emerging epidemic has been observed for HNSCC at subsites associated with HPV, while a decline or stable trend for most HPV-unrelated HNSCC subsites has been recorded,^4^ possibly reflecting the reduction of tobacco and alcohol consumption. Yet, the increasing incidence of HPV-negative oral cavity squamous cell carcinomas (OCSCC) has been observed in non-smoking and non-drinking patients in several countries, involving especially the oral tongue.^5–8^ The incidence increase was observed especially in young adults, and more frequently in young women.^9–13^ Moreover, an increased incidence of SCC of the gingiva and the hard palate has been reported among older patients, mostly women, with no history of tobacco or alcohol use.^14^ Neither HPV infection nor current trends in tobacco and alcohol consumption could explain these rising incidence rates, suggesting the possibility of exposure transitions involving unknown carcinogenic factors. Moreover, distinct clinico-pathological features have been observed in young, non-smoking patients with OCSCC, compared to their smoking counterparts, including a higher incidence of regional cancer recurrence.^15,16^

Emerging evidence indicating that these OCSCC tumors can be a new, specific clinical entity warrants a better understanding of their associated etiological factors and molecular characteristics. Detailed molecular characterization of OCSCC in patients with no identified risk factors (hereafter referred to as NIRF) will contribute to deciphering the underlying molecular mechanisms as well as refine the histopathological classification of cancers of oral cavity and, in particular, oral tongue cancers with possible implications for improved diagnosis and treatment.

Mutation landscapes of cancer genomes result from an imbalance between the DNA damage by exogenous or endogenous factors and the efficiency of the DNA repair system.^17^ The mutagenic process-specific patterns, so-called mutational signatures, can be identified by a blind source separation-based mathematical process that deconvolves mixed mutation spectra.^18–20^ Comprehensively identified mutational signatures can thus reveal the DNA-damaging processes operative during a cancer’s history. Reports on various HNSCC cohorts aimed to decipher diverse mutational processes, identifying the clock-like mutagenesis (COSMIC signatures SBS1 and SBS5),^19,21–25^ mutagenesis by APOBEC enzymes (SBS2, SBS13),^19,21–26^ tobacco smoking and putative alcohol exposure (SBS4, SBS16),^19,22–25^ DNA repair deficiency (SBS3, SBS6),^19,21–23^ DNA damage by reactive oxygen species (SBS18),^19,24,25^ and various mutational signatures of unknown origin (SBS17, SBS33, SBS40).^19,25^

Tobacco smoking plays a strong role in laryngeal cancer risk, whereas alcohol - even in smokers - is a weaker risk factor than tobacco. Typically, only the upper part of the larynx becomes directly exposed to alcohol.^27^ Therefore, in contrast with OCSCC, no excess risk is observed in patients with laryngeal cancer with moderate alcohol intake.^27^ Yet, only a few studies analyzed the mutational signatures according to the specific HNSCC subsites.^19,23,26,28^ Alexandrov et al. and South et al. reported differences between laryngeal SCC (LXSCC) and OCSCC with regard to the tobacco smoking signature (SBS4).^24,25^ Some reports noted varying rates of the endogenous signature SBS5 between smokers and non-smokers, yet none have identified mutational processes operating specifically in the NIRF patients with OCSCC.^24,25,29,30^ Lastly, with the exception of the APOBEC mutational signature, which has been reported to correlate with the HPV infection,^22,26,28^ none of the signatures identified to date have been associated with specific clinical presentations or cancer progression.

Here we performed an in-depth analysis of mutational signatures in HPV-unrelated subsites, i.e., LXSCC and OCSCC, in order to characterize relevant mutational processes and driver gene alterations underlying the trajectories of their development.^31^ Using innovative analysis of complex molecular cancer data from public sources, we particularly focused on the molecular characterization of OCSCC in NIRF patients, including early onset tongue cancers as well as OCSCC in older adults. LXSCC were included as tobacco-smoking-related controls. We further investigated clinically relevant stratification of the studied OCSCC and LXSCC subsites according to the most active mutational processes supported by specific epigenetic and transcriptomic programs, to gain a new understanding of the biological underpinnings of their development. Furthermore, we investigated exposure- and anatomical site-specific molecular programs to define novel aspects for the roles of alcohol drinking and tobacco smoking. Collectively, our results provide new insights into the molecular landscapes of NIRF OCSCC, an emerging yet understudied clinical entity with rising incidence.

## Results

### Patient clustering based on de novo mutational signatures

**Fig. 1:**
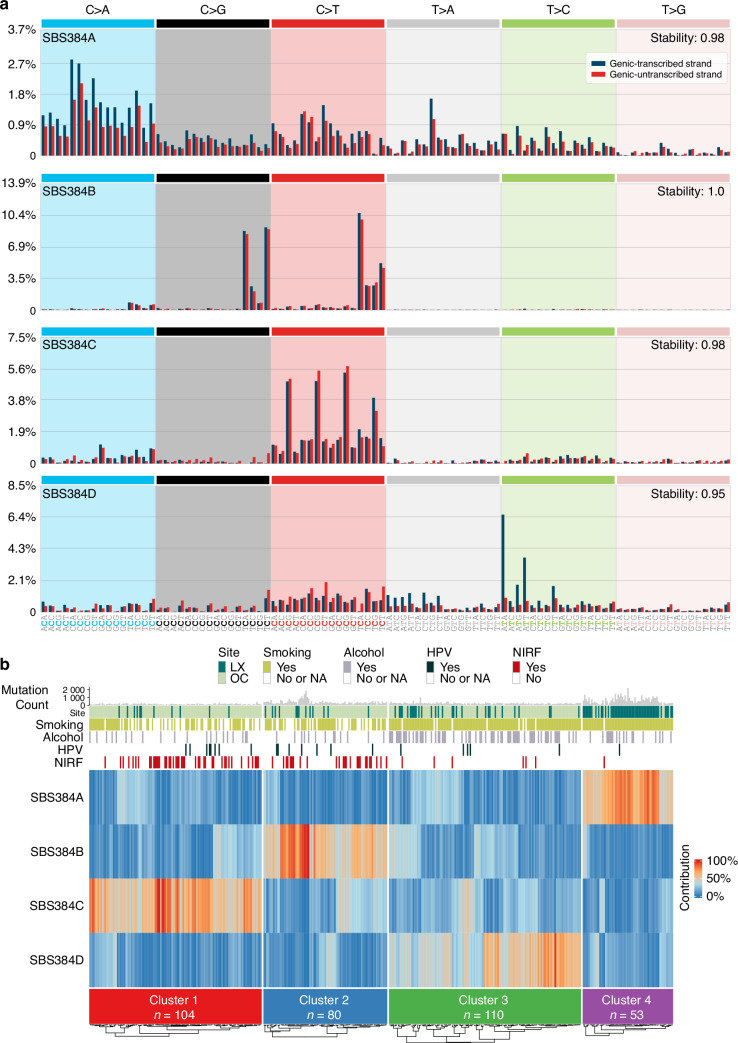
Mutational signatures characterizing the major OCSCC and LXSCC subsets. **a** De novo transcriptional strand-asymmetry signatures (signatures SBS384A through SBS384D extracted by NMF, see Methods). **b** De novo signatures determine four major patient clusters. The heatmap is based on the relative per-sample contribution of each mutational signature. Heatmap annotation tracks and color legends: Mutation count histogram, anatomical subsites (LX = larynx, OC = oral cavity), tobacco smoking status, alcohol use, HPV infection status, and no identified risk factors (NIRF)

Following initial sample filtering (see Methods, Supplementary Figs. S1 and S2), mutational signature analysis of a total of 347 (253 (73%) OCSCC and 94 (27%) LXSCC) whole-exome sequenced (WES) samples was performed using the 384-channel (i.e., transcription strand-biased) signature versions to enhance sensitivity. This analysis identified four distinct de novo signatures SBS384A-D (Fig. 1a). Their relative per-sample contribution was used to group the samples into four distinct clusters (Fig. 1b), with each cluster characterized by the prevailing activity of one de novo signature, in terms of absolute mutation counts (Supplementary Fig. S2d). Signature SBS384A was significantly more prevalent in the fourth cluster (*n* = 53) compared to the three other clusters. Similarly, signature SBS384B was significantly enriched in the second cluster (*n* = 80), SBS384C in the first cluster (*n* = 104), and SBS384D in the third cluster (*n* = 110) (Fig. 1b).

To further characterize the individual de novo signatures, which often represent mixed patterns, we performed their decomposition into the COSMIC reference signatures. Using a tailor-designed signature assignment approach (see Methods and Supplementary Fig. S3–S6), we identified a final set of six COSMIC signatures to be the most prominent (Supplementary Fig. S4c). The clock-like SBS1 signature dominated cluster 1 (renamed SBS1 cluster). In cluster 2, both APOBEC-related signatures, SBS2 and SBS13, were detected along with elevated SBS1 activity (renamed the SBS1/APOBEC cluster). SBS16, a signature previously associated with alcohol consumption, dominated in cluster 3 (renamed the SBS16 cluster). Lastly, SBS4 and SBS92, two signatures linked to tobacco smoking, were exclusively detected in cluster 4 (renamed the SBS4/SBS92 cluster) (Supplementary Fig. S7).

### Clinico-pathological characteristics match the mutation-signature clusters

**Fig. 2:**
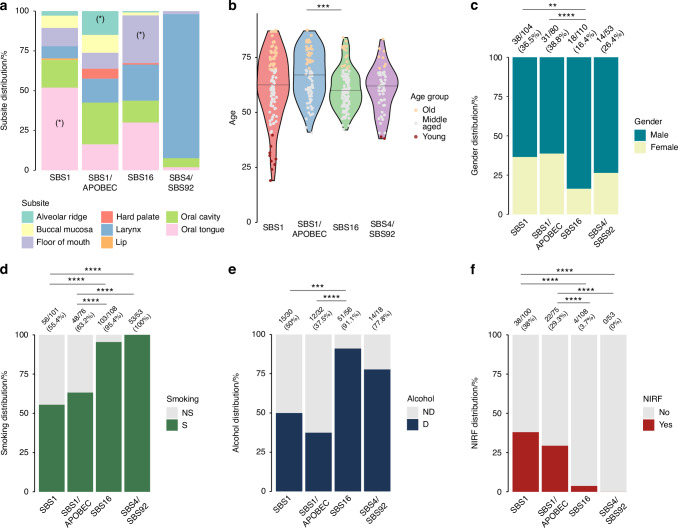
Mutational signature cluster-based demographic and clinical presentation summaries. **a** Relative anatomical subsite distribution in each cluster, all patients. (*) Significant in any 2-cluster comparison (all comparisons are available in Supplementary excel file 1). **b** Distribution of patients by age (young patients ≤40; 40<middle aged<70; old ≥70) per cluster. Median age (yr): SBS1 = 61.9, SBS1/APOBEC = 66.9, SBS16 = 60.7, SBS4/SBS92 = 60.1. **c** Relative gender-based patient distribution in each cluster **d** tobacco smoking status and **e** Alcohol consumption distributions across clusters. **f** NIRF status across clusters. NA status not plotted. Significance indicated by asterisks: *P < 0.05, **P < 0.01, ***P < 0.001, ****P < 0.000 1. When present, the ratios and percentages on top represent the proportions of the bottom bars

The signature-based clusters differed significantly regarding anatomical subsite involvement (Fig. 2a and Supplementary Fig. S8), age distribution (Fig. 2b), sex (Fig. 2c), smoking (Fig. 2d) and alcohol use history (Fig. 2e). Oral tongue SCC (OTSCC) (*n* = 54/101, 53%) were mainly observed in SBS1 cluster and represented the majority of cancers in that cluster (n = 54/104, 52%). The remaining OTSCC were distributed between the SBS16 cluster (*n* = 33/101, 33%), the SBS1/APOBEC cluster (*n* = 13/101, 13%), and the SBS4/SBS92 cluster (*n* = 1/101, 1%). Floor of mouth tumors were mainly (*n* = 33/54, 61%) in the SBS16 cluster, while the majority of tumors involving the hard palate (*n* = 5/6, 83%) and the alveolar ridge (*n* = 12/16, 75%) were clustered in the SBS1/APOBEC cluster. Tumors affecting the buccal mucosa were detected in the SBS1 (*n* = 8/19, 42%) and SBS1/APOBEC (*n* = 9/19, 47%) clusters. Overall, OCSCC represented 92% (96/104), 85% (68/80) and 70% (77/110) of samples in the SBS1, the SBS1/APOBEC, and the SBS16 clusters, respectively, while the LXSCC represented 94% (48/53) of samples in SBS4/SBS92 cluster (containing 52% (48/93) of all LXSCC). The remaining LXSCC were dispersed mainly in the SBS16 and the SBS1/APOBEC clusters.

Eight of the ten young patients (<40 years) were in the SBS1 cluster (*n* = 8/10, 80%), while the majority of the elderly patients (≥70 years) were equally distributed between the SBS1 and SBS1/APOBEC clusters (*n* = 34/91, 37% and *n* = 32/91, 35%, respectively) (Fig. 2b). While a higher proportion of the female patients (*n* = 69/101, 68%) were clustered in the SBS1 and SBS1/APOBEC clusters (*n* = 38/101, 37% and *n* = 31/101, 31%, respectively), males were nearly equally distributed between the SBS1 and SBS1/APOBEC clusters and the SBS16 and SBS4/SBS92 clusters (*n* = 115/246, 47% and *n* = 131/246, 53%, respectively) (Fig. 2c).

Among patients with documented tobacco use, 60% (156/260) were present in the SBS16 and the SBS4/SBS92 clusters, made up of 95.4% (103/108) and 100% (53/53) tumors of smokers, respectively (Fig. 2d).

The majority of tumors from the patients with documented alcohol consumption (n = 65/92, 70%) were present in the SBS16 and the SBS4/SBS92 clusters that were respectively composed of 91% (51/56) and 78% (14/18) of tumors of drinkers (Fig. 2e).

Of note, the large majority of the NIRF OCSCC (*n* = 60/64, 94%) were present in the SBS1 and SBS1/APOBEC clusters (*n* = 38/64, 60% and *n* = 22/64, 34%, respectively) (Fig. 2f).

The distribution of clinical stages did not differ significantly between clusters (*P* = 0.136). Overall survival approached significance (*P* = 0.06) across clusters, whereas disease-free survival remained non-significant (*P* = 0.44) (Supplementary Fig. S9).

### NIRF-enriched clusters are driven by endogenous mutagenic processes, while tobacco smoking-related clusters reveal site-specific mutational processes

**Fig. 3:**
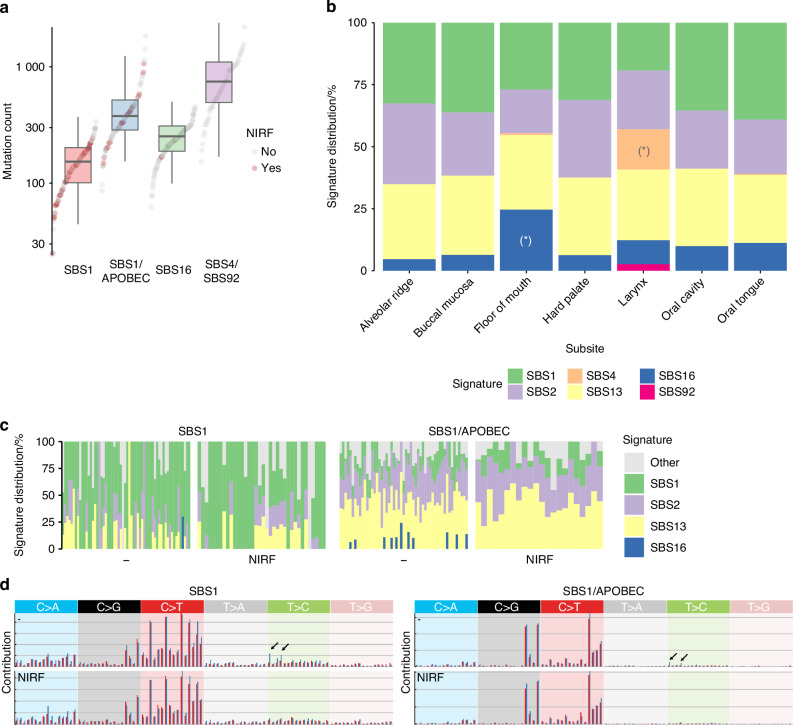
Tobacco exposure impact. **a** Per-sample and per-cluster distributions of the total mutation counts (median and range). **b** Relative distribution of samples positive for either of the main six SBS signatures according to subsite. (*) Significant differential enrichment (all comparisons are available in Supplementary excel file 1). **c** Relative distribution of SBS16 signature across smokers/drinkers (-) and NIRF patients. **d** Mean profile spectra of NIRF and non-NIRF patients in NIRF-enriched clusters (SBS1 and SBS1/APOBEC)

NIRF-enriched clusters were characterized by increased levels of signature SBS1 in terms of both relative and absolute mutation counts (Supplementary Figs. S4a, S7). Moreover, APOBEC signatures SBS2 and SBS13 were predominant in the SBS1/APOBEC cluster (Supplementary Figs. S7, S4a and S4c). The mutation burden also differed significantly between the two NIRF-enriched clusters (SBS1, median = 139, range = 24–338, and SBS1/APOBEC, median = 344, range = 86–1 835), likely due to the activation of the highly mutagenic APOBEC enzyme (Fig. 3a).

Interestingly, the mutagenic impact of smoking exposure varied across clusters. The mutation burden was higher in the SBS4/SBS92 cluster (median = 682, range = 153–2 193) compared to the SBS16 cluster (median = 230, range = 62–457) (Fig. 3a), despite both being enriched for smokers (Fig. 2d). These differences could be related to the tumor site as SBS4 and SBS92 were almost exclusively detected in the highly mutated SBS4/SBS92 larynx-enriched cluster (Fig. 3b). SBS16 was found in smokers’ SCC and at higher levels in SCC of smokers who were also drinkers (Supplementary Fig. S10). This suggests that tobacco smoke has selective, anatomical site-specific mutagenic effects, with distinct mutational processes occurring in the laryngeal tissues of smokers (SBS4, SBS92) and in oral cavity tissues (SBS16).

In fact, analysis performed on 500 synthetic samples revealed mutation count-dependent over-attribution of SBS5 by SigProfilerExtractor tool (see Materials and Methods and Supplementary Fig. S6), potentially masking signatures SBS16 and SBS92 in the TCGA data. Limiting the SBS5 overfitting using the Mutational Signature Analysis (MSA) tool (see Materials and Methods), which provides simulation-based confidence intervals without making prior assumptions about signatures, facilitated the identification of SBS16 in the OC (enriched in cluster SBS16) and made SBS92 clearly discernible in tobacco smoking-associated laryngeal cancers (cluster SBS4/SBS92)(Supplementary Fig. S4a, c).

On the other hand, tumors from smokers/drinkers found in the SBS1 and SBS1/APOBEC clusters rarely contain any tobacco-related signature, with SBS16 only detected in few samples (Fig. 3c). Consequently, NIRF and smoking/drinking patients shared similar mean mutation spectra in these clusters, except for the low presence of transcription strand biased T > C mutation pattern in tumors from smoking/drinking patients (Fig. 3d). This low presence of signature SBS16 suggests a minimal contribution of alcohol and tobacco to the total mutation load in these tumors, which may indicate that these cancers may share with NIRF cancers other, yet to be determined etiological factors.

In sum, NIRF OCSCC are defined by elevated mutagenic activities of endogenous processes that result in the enriched presence of COSMIC signatures SBS1 and SBS2/13.

### Cancer driver gene mutations in the NIRF-enriched clusters

**Fig. 4:**
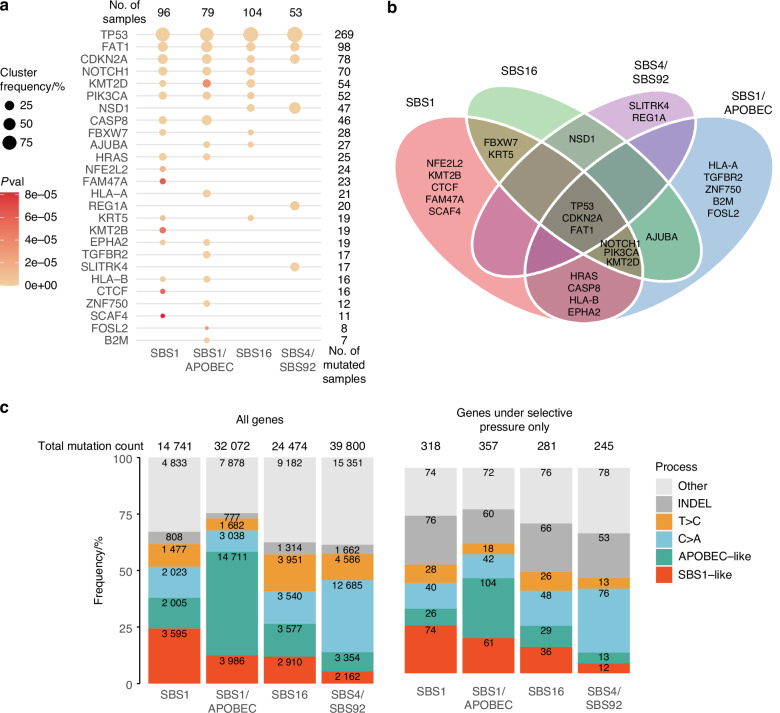
Cluster distribution of non-silently mutated genes under selective pressure, identified by the non-synonymous/synonymous (dN/dS) mutation ratio approach. **a** Summary of the mutated genes under selective pressure identified by the dN/dS analysis (see Materials and Methods). The number of samples in each cluster is shown in the top row, and number of cases with a mutated gene (with symbols listed on the left) is shown on the right. *p*-values for substitutions were obtained by Likelihood-Ratio Tests as described^84^ and cluster frequency indicate percentage of the samples in each cluster mutated for a given gene. **b** Venn diagram showing the distribution of mutated genes under selective pressure across clusters. **c** Relative distribution of signature-matched mutation processes for all mutated genes in each cluster (left) and mutated genes that are under selective pressure (right). Numerical values in the graphs indicate the underlying mutation counts

The presence of distinct mutational processes predominantly operating in each cluster suggests potential differences in disease etiology and development. To test this hypothesis, we determined mutated genes under selective pressure by using the non-synonymous/synonymous (dN/dS) mutation ratio approach (see Materials and Methods). This analysis revealed 26 mostly recurrently mutated genes under positive selection with the following distribution: SBS1 cluster – 18/26; SBS1/APOBEC cluster – 17/26; SBS16 cluster – 11/26; SBS4/SBS92 – 6/26 (Fig. 4a).

The mutated tumor suppressor genes *TP53*, *CDKN2A,* and *FAT1* were selected in all clusters, whereas various mutated epigenetic modifiers under positive selection exhibited more cluster-specific distributions. *KMT2B* mutations were only detected in the SBS1 cluster, whereas mutations in the histone methyltransferase *NSD1* were restricted to the tobacco smoking clusters (SBS4/SBS92 and SBS16). Interestingly, genes involved in mitogen-activated protein kinase cascade components (*HRAS* and *EPHA2*) were mutated and selected only in the NIRF-enriched clusters. An altered antigen presentation system (mutated *HLA-A*, *HLA-B, B2M genes*) and immune cytotoxic response (mutated *CASP8 gene*) were also specific to the NIRF-enriched clusters (SBS1/APOBEC and SBS1), suggesting the presence of immune pressure in these clusters (Fig. 4b).

### The functional impact of mutational signatures

To address the impact of the key mutational signatures on all mutated genes, we selected the substitution sequence contexts that were most representative of a given mutagenic process and examined their presence in the mutated genes. The APOBEC (APOBEC-like) mutational process was represented by T[CW (where W = A or T), and the SBS1 was represented by X[C > T]G (X = either of A, T, C, G). As expected, gene mutations associated with the APOBEC-like pattern were more frequent in the SBS1/APOBEC cluster, while gene mutations resembling the SBS1-like pattern were also more prevalent in both the SBS1/APOBEC and SBS1 clusters (Fig. 4c). Furthermore, the C > A gene mutations (dominant in SBS4) were enriched in the SBS4/SBS92 cluster and the T > C mutations in the SBS16 cluster. While we observed that the selected genes were relatively more enriched for small insertions/deletions (indels) compared to all mutated genes (Fig. 4c), the distribution of mutations was nearly identical for both, all mutated genes and the subset of genes under selective pressure (Fig. 4c). This suggests that the overall processes mutagenizing single bases identified for each cluster were also affecting the genes selected during cancer development.

### NIRF-enriched clusters exhibit gene expression programs of antimicrobial responses and keratinization

To gain deeper functional and biological insights into the HNSCC patient stratification based on mutational signatures and to further assess the robustness of this approach, we performed DNA methylation analysis, differential gene expression, and pathway analysis of the available TCGA methylome and transcriptomic data.

**Fig. 5:**
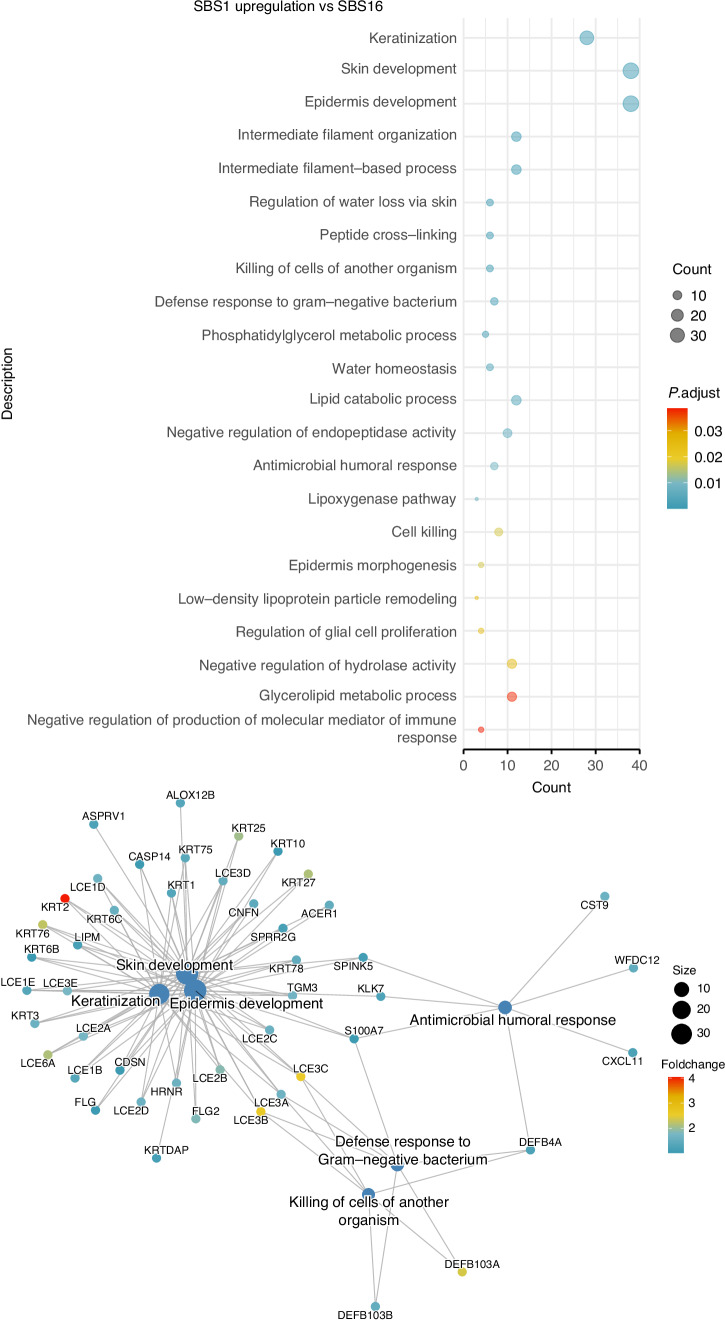
Enrichment of the clusters for functional biological processes. Pathway enrichment for genes differentially expressed in the SBS1 cluster using the cluster SBS16 as reference. Top panel: Pathways with enrichment *P*-values < 0.05 are listed on the left alongside count indicators representing the number of genes enriched in each pathway. Bottom panel: Network diagram shows selected top pathways of interest and their respective differentially upregulated genes

**Fig. 6:**
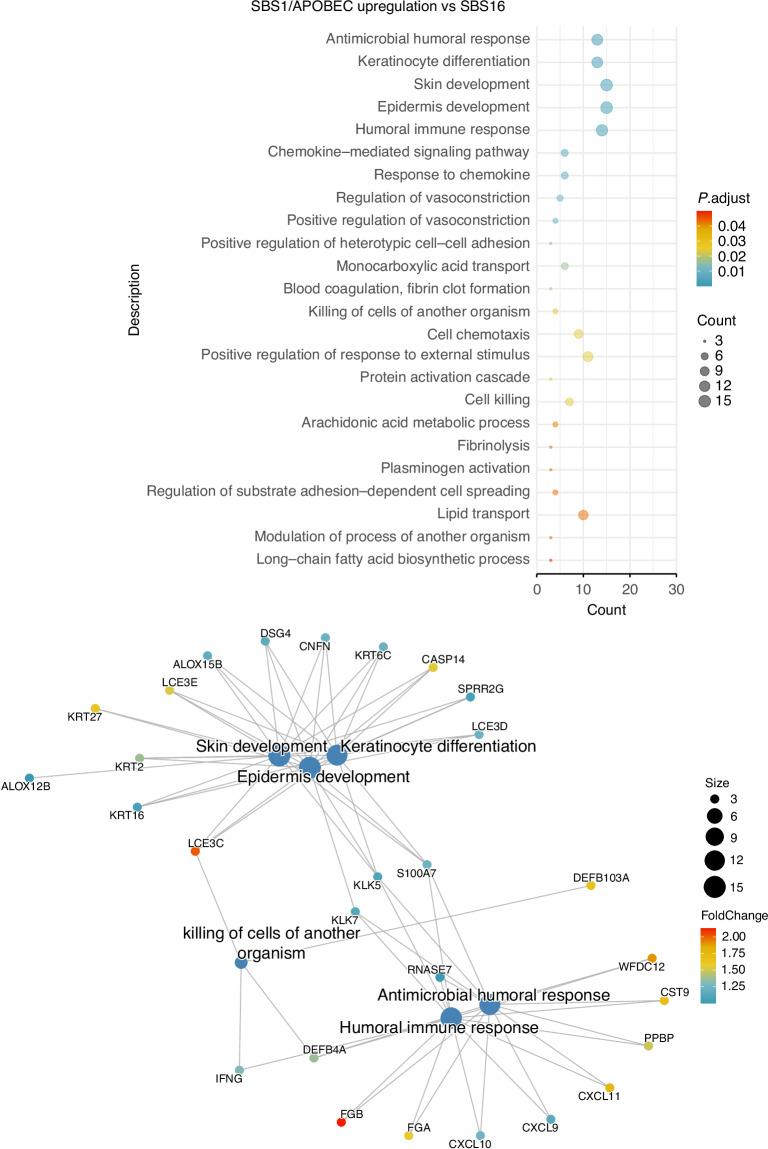
Enrichment of the SBS1/APOBEC cluster for functional biological processes. Pathway enrichment for genes differentially expressed in the SBS1/APOBEC cluster using the cluster SBS16 as reference. Top panel: Pathways with enrichment *P*-values < 0.05 are listed on the left alongside count indicators representing the number of genes enriched in each pathway. Bottom panel: Network diagram shows selected top pathways of interest and their respective differentially upregulated genes

By investigating the distribution of the above methylation patterns in each cluster, we showed that most patients in the two NIRF-enriched clusters harbored atypical, non-smoker DNA methylome patterns (Supplementary Fig. S11). Moreover, the NIRF-enriched clusters characteristically exhibited increased antimicrobial humoral response pathway activation (involving the genes *S100A7*, *KLK7*, *CXCL11*, *CST9*, *WFDC12*, and *DEFB4A*) and higher expression of genes involved in the keratinization pathways (skin/epidermis development, keratinization, keratinocyte differentiation) (Figs. 5 and 6). In contrast, such activation of the keratinization pathway was absent in the tobacco smoking cluster SBS4/SBS92 (Supplementary Fig. S12).

To validate the in silico findings, we evaluated the intratumor distribution of relevant markers in two OTSCC cases: one NIRF case and one from a non-smoking, HPV-negative light drinker (Supplementary Fig. S13). We assessed pan-cytokeratin as a readout of keratin upregulation and S100A7 as a marker of antimicrobial response. Both tumors showed high keratin expression, with prominent pancytokeratin staining at the invasive front. S100A7 staining was present in a non-homogeneous manner across the tumor. Of note, both tumors were also positive for punctate lipopolysaccharide (LPS) signal (Supplementary Fig. S13), suggesting a possible involvement of microbial components and warranting further investigation of microbial populations and host responses in oral squamous cell carcinoma.

**Fig. 7:**
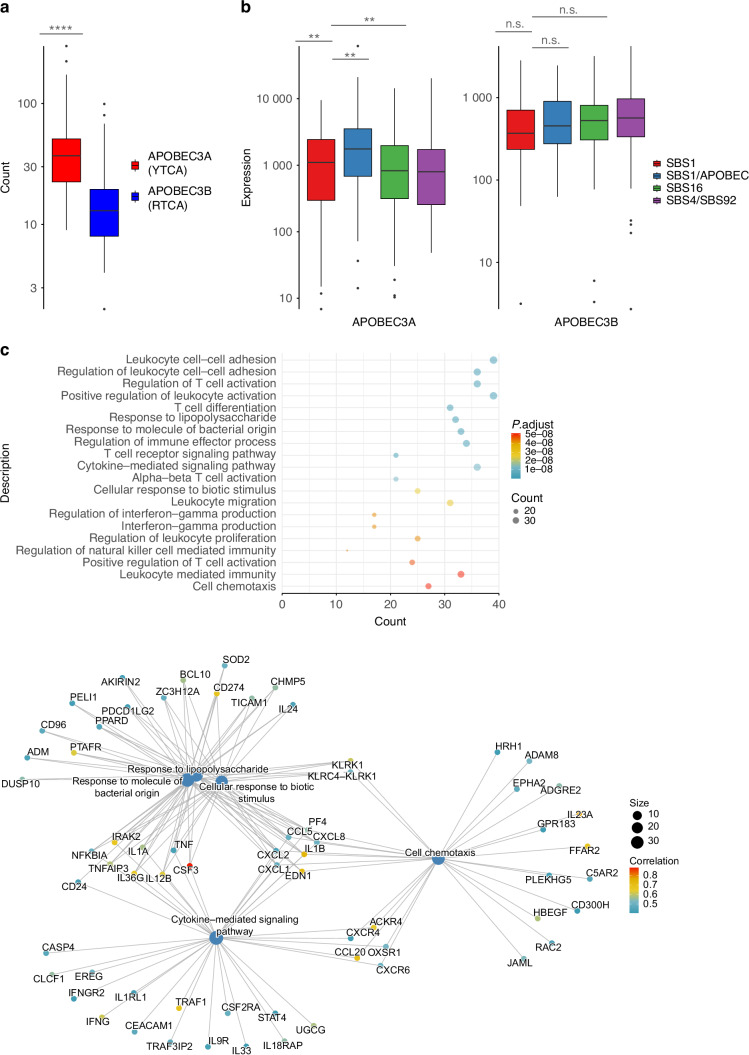
Origin of APOBEC mutations. **a** mutation count in SBS1/APOBEC cluster for APOBEC3A (YTCA, Y replacing a pyrimidine base) and APOBEC3B (RTCA, R replacing a purine base) targeted specific context and. **b** APOBEC3A and APOBEC3B expression across clusters. **c** Top 20 pathways enriched for genes correlated with APOBEC3A expression. Significance indicated by asterisks : *P < 0.05, **P < 0.01, ***P < 0.001, ****P < 0.000 1. Bottom panel: Selected top pathways of interest and their respective differentially upregulated genes

We next determined whether the activation of APOBEC enzyme activity revealed by the APOBEC-directed mutagenesis is associated with a specific immune status of patients in the NIRF-enriched clusters. APOBEC-induced mutations in cancer have been shown to be generated by the APOBEC3A and APOBEC3B enzyme, with distinct isoform-specific substrate preferences.^32^ APOBEC3A preferentially targets YTCA sequences (Y = pyrimidine bases) whereas APOBEC3B is more prone to mutate RTCA sequences (R = purine bases). We analyzed the APOBEC mutation context and observed a higher APOBEC3A activity marked by the enriched YTCA context in the SBS1/APOBEC cluster (Fig. 7a). The APOBEC3A mRNA expression was elevated in the SBS1/APOBEC cluster indicating that APOBEC3A remained active at the time of tumor sampling (Fig. 7b). Interestingly, the APOBEC3A expression in the SBS1/APOBEC cluster correlated with activated genes and pathways involved in the bacteria-specific immune response (Fig. 7c). Immune cell deconvolution further showed that the SBS1/APOBEC cluster differed significantly from the SBS4/SBS92 group, with higher proportions of proinflammatory M1 macrophages and CD8+ T cells (Supplementary Fig. S14).

In sum, the mutational signature-based patient clustering coupled with gene regulation/expression analysis suggest a possible etiology of the NIRF OCSCCs associated with cell keratinization and immune responses against bacterial insults.

## Discussion

Mutational*-*signature*-*based clustering of 253 OCSCC and 94 LXSCC, used as tobacco-related controls, allowed to discern distinct molecular programs of previously unrecognized OCSCC subtypes with rising incidence. While stage distribution and overall or disease-specific survival did not significantly differ between clusters, the present results support differences in the etiology and development of early-onset mobile tongue SCC, currently a poorly characterized clinical entity of increasing incidence. We also established that OCSCC and LXSCC can be stratified according to anatomical subsite-specific mutational signatures and distinct sets of recurrently mutated, selected-for driver genes, reflecting different carcinogenesis processes. Our comprehensive analysis did not reveal mutational signatures suggestive of specific environmental mutagens operative in the NIRF-enriched patient subgroups.

By excluding samples with rare mutation spectra to prevent signature bleeding and incorporating confidence intervals during signature fitting, our approach allows for improved OSCC and LXSCC signature attribution.

We found that SBS16 mainly manifested in the OC tumors of smokers, especially those localized in the floor of the mouth. The signature SBS16 has been previously proposed to be linked to alcohol consumption.^33–37^ Yet, it was also observed in tumor and non-tumor tissues of patients without previous history of alcohol exposure^33,35–37^ and in HNSCC linked to tobacco smoking.^38^ In keeping with previous studies showing that SBS16 can form in the absence of substantial alcohol exposure history,^39–43^ SBS16 was found in smokers’ SCC with higher levels in SCC of smokers-drinkers. Of note, none of the 12 OC of non-smoking drinkers harbored signature SBS16. This suggests that in HNSCC, signature SBS16 forms mainly due to the combined effects of tobacco smoking and alcohol drinking, or smoking only, rather than to alcohol exposure alone. This is in agreement with previous observations made in independent analyses and cohorts, Plath et al.^23^, Gillison et al.^22^ and Torrens et al.^38^ as well as epidemiological evidence reporting that the joint effect of tobacco and alcohol on HNSCC risk is greater than multiplicative.^44^ Additional support for the association between tobacco smoking and SBS16 has been recently documented by a study on somatic mutagenesis in the non-cancerous bronchial epithelium.^45^

Thus, alcohol and tobacco might elicit the same mutational process or synergize to drive mutagenesis manifesting primarily in the floor of the mouth. Alexandrov et al. reported that tobacco consumption can increase the rates of SBS5 mutagenesis in OC^25^ yet given the similarity between SBS16, SBS5 and also SBS92 (Supplementary Fig. S5) and the by-default inclusion of SBS5 in the SPE-based decomposition process, a role for overfitting of SBS5 is likely and should be considered. It should be noted that the analysis reported here is based on whole-exome sequencing with some samples harboring lower mutation counts, especially in the SBS1 and SBS16 clusters. Lower mutation counts can make the conditions for signature extraction less optimal, potentially affecting the diverse outcomes observed upon the SPE signature extraction and MSA signature assignment due to SBS5 overfitting.

We next observed that in tobacco smokers, the mutational processes and the overall mutation burdens differed between the OC and the LX, with the median mutation count nearly 10-fold higher in the LX. The mutational signatures SBS4 and (to a lesser extent) SBS92, both related to tobacco smoking were the main contributors to the mutational landscapes of the laryngeal tumors, yet they were barely detectable in the OC cancers of smokers.

Site-specific differences in mutation patterns in patients exposed to the same carcinogens might be due to the anatomical subsite susceptibility or protective capacity. For example, saliva has been shown to curb mutagen activity,^46–50^ a property that can explain the lower mutation burden observed in the OC. Moreover, mutagen concentration during the oral preparatory stage of swallowing,^51^ when the bolus is held in the anterior part of the floor of the mouth, could explain the higher proportion of SBS16 at that subsite. However, the precise molecular mechanisms underlying the signature SBS16 formation warrant further investigations.

NIRF OCSCC were detected in the SBS1 and SBS1/APOBEC clusters, both of which are associated with two endogenous mutational processes targeting cytosine residues. The SBS1 cluster was dominated by the activity of mutational signature SBS1, consistent with a previous finding reported in the literature.^23^ SBS1 is enriched for [C > T]pG mutations and it arises from endogenous processes, including spontaneous deamination of methylated cytosines, or enzymatic effects such as those exerted by the APOBEC cytidine deaminase family.^52–54^ The resulting T:G mismatch can be corrected by the DNA mismatch repair system before DNA replication. Consequently, the SBS1 mutation pattern can accumulate in the stem cells over the course of lifetime in relation with the frequency of their division, approximately reflecting the advancement of age.^55^

As a clock-like signature, SBS1 is expected to be ubiquitous nevertheless its absolute and relative increase within the SBS1 NIRF-enriched cluster was somewhat surprising as patients in that group tend to be younger on average than the other HNSCC patients. Since all samples analyzed were fresh frozen, FFPE-related artifacts known to resemble SBS1^56^ can be ruled out. The observed variation in SBS1 levels is therefore interpreted as biologically driven, potentially reflecting enhanced spontaneous deamination of 5-meC, increased cell proliferation,^57^ or replication-independent mechanisms.^58^ Notably, replication errors introduced by DNA polymerase ε have also been proposed to contribute to SBS1-like patterns.^59^ However, this latter mechanism is likely limited in our cohort given the low mutation rates observed in *POLE* and other DNA polymerase genes (unpublished results). It remains to be established whether and to what extent either of these mutagenic processes are involved in the development of NIRF OCSCC.

Our study also uncovers distinct cancer development trajectories, as evidenced by the recurrently mutated genes under positive selection, which have important clinical implications. While the selected-for mutations in the tumor suppressor genes *TP53*, *CDKN2A* and *FAT1* were observed in all patient-signature clusters, mutated epigenetic modifiers differed between the individual clusters. The recurrently mutated histone lysine methyltransferase *NSD1* was only detected in clusters enriched with smokers (SBS16 and SBS4/SBS92) irrespective of the cancer subsite, suggesting that mutated *NSD1* could release a constraint imposed by tobacco exposure.

These findings align with previously published studies,^60–62^ which identified distinct methylation-driven programs including one related to a hypo-methylated subgroup harboring NSD1 or histone H3K36M alterations.^61^ *NSD1*-deficient tumors have been described as immune-cold tumors, with lymphocytes restricted to the stroma of the tumor borders, failing to infiltrate the tumor.^63^ *NSD1* inactivation drives tumor evasion^63–65^ by reducing interferon-stimulated gene expression. Conversely, restoring methylation with the EZH2 histone-lysine N-methyltransferase inhibitor re-establishes immune response,^63^ offering a potential therapeutic strategy for patients with NSD1 inactivation mostly present in the SBS4/SBS92 cluster and also in the SBS16 cluster. Further multi-omic analyses will be essential to better understand how these epigenetic alterations shape transcriptional programs that influence tumor development and immune activity.^66,67^

Alternatively, SBS1 and SBS1/APOBEC clusters where enriched in two “atypical” subtypes: one characterized by methylated CpG islands and the other lacking them described by Brennan et al. ^62^. It remains unclear whether the two NIRF-enriched clusters represent two distinct disease types, as is suggested by varying distribution of affected subsites and involvement of distinct age groups, or the same clinical entity with a subset characterized by overactivation of the APOBEC3A enzyme and related mutagenesis. In support of the latter, we observed that both NIRF-enriched clusters harbor higher SBS1 mutation counts, and both clusters appear to have acquired a specific immune escape response, as indicated by the positive selective pressure on shared mutated genes involved in Class I MHC antigen presentation system (*HLA-B*, *HLA-B, B2M*) and immune cytotoxic response (*CASP8*) suggesting the presence of a strong immune cytolytic control mechanism.^68^ The specific immune status of both NIRF-enriched clusters is further underscored by overlapping differential gene expression profiles showing the presence of bacteria-specific epithelial and immune responses. Recent findings on intratumoral and intracellular bacteria invading the tumor cells offer an attractive putative explanation of the observed distinctive immune status.^69–72^ The immune evasion strategies identified in NIRF OCSCC may have important clinical implications. These findings suggest that inhibitors targeting the PD1 axis will have limited effects on the NIRF SCC tumors as defective antigen presentation is a source of primary or acquired resistance to immune checkpoint inhibitors.^73^ In addition, APOBEC3A overexpression points to a distinct source of replication stress and genomic instability in this cancer subtype. Such a molecular context may create a selective susceptibility to ATR-targeted therapies, offering a potentially more precise treatment option for these patients.^74^

Although APOBEC activation in HNSCC has been previously linked to HPV infection,^75^ HPV positivity in the SBS1/APOBEC cluster was rare, with only 7.5% of cases testing positive. Other viral origins of OCSCC in non-smoker and non-drinker patients have been proposed.^76^ In our study, APOBEC3A expression correlated with the antibacterial responses which may represent an alternative point of departure for APOBEC activation. However, if the cancers in the SBS1 and SBS1/APOBEC clusters are different manifestations with a common origin, it remains to be understood why patients in the SBS1/APOBEC group, in addition to APOBEC activation, present with distinct OCSCC localizations at an older age.

The antimicrobial response observed in SBS1 and SBS1/APOBEC warrants further investigations. The tumor regions displaying localized overexpression of cytokeratin and S100A7 suggest a heterogeneous distribution of these markers, alongside some localized bacterial positivity. This aligns with previous reports of intratumoral and intracellular presence of bacteria in cancers, including OCSCC,^70,72,77,78^ possibly contributing to tumor development through inflammatory, signaling, mutagenic and metastatic pathways^79^ However, the relationship between bacterial presence and tumor biology is subject to vigorous debate^80,81^ and future comprehensive analysis of the oral microbiome’s composition and role in OCSCC tumorigenesis is warranted.

## Conclusion

Our study represents the first molecular and genomic characterization of NIRF OCSCC revealing distinct molecular programs of this emerging pathological entity with increasing global incidence. Stratifying HNSCC by mutational processes using public repository data offers a powerful framework for uncovering new insights into this emerging subtype. Although conducted in a U.S. cohort, this analysis provides a solid basis for similar investigations in other geographical regions to assess the general relevance of the findings.

Moreover, our study refines the molecular understanding of HPV-unrelated HNSCC, reclassifying tobacco-related cases as site-specific clinical entities with unique tumorigenic pathways. The distinctive mutational signatures identified in the NIRF OCSCC, exhibiting immune evasion strategies, antimicrobial responses, warrant further investigations into the etiology and clinical implications.

## Materials and Methods

### Samples and clinical data

TCGA Clinical data (528 patients) were downloaded from the Genomic Data Commons [GDC release v33.0 downloaded on 2022/05/11]. Clinical, demographic and lifestyle variables (cancer subsite, age, sex, ethnicity, tobacco smoking, alcohol consumption) for all available whole exome sequenced (WES) samples were extracted and are described in Supplementary excel file.

After excluding 103 samples corresponding to the hypopharynx and oropharynx, the dataset included 425 samples, i.e., 314 OCSCC samples and 111 LXSCC s, with the latter serving as a tobacco smoking-related control group.

Cases were classified into three age groups (young ≤ 40 years, middle-aged 41-69 years and old ≥70 years). Tobacco-smoking status was classified as lifelong non-smoker (less than 100 cigarettes in the lifetime) or ever smoker. Alcohol consumption status was classified as non-drinker (less than 1 drink/day) or ever-drinker. HPV infection status was obtained through the study of Campbell et al.^82^. Briefly, HPV status was assessed independently for all samples in two different centers by two different techniques (DNA sequencing and PathSeq algorithm and RNA-seq expression levels). Cases were classified as NIRF if they were non-smokers, non-drinkers, and HPV-negative. A fraction of the analyzed cases (8 of 425, or 1.9%) had undergone neoadjuvant treatment making it unlikely to confound the reported overall mutation burden and mutational signature analyses.

To validate select in silico results, two early-stage OTSCC cases, OTSCC #1 (age 45) and OTSCC #2 (age 40) were processed for histopathologic analysis. Both were HPV-negative White non-smokers with lateral tongue tumors. Clinical data included tumor stage, p16 and HPV status, recurrence, follow-up, and survival. One had locoregional recurrence but both were alive with no disease at last follow-up. Standard clinical and pathological protocols were followed for evaluation. The use of the patient tumor material was approved by the University of Wisconsin-Madison’s Minimal Risk Institutional Review Board (Study No. 2018-1510), and by the International Agency for Research on Cancer Ethics Committee (Project IEC 25-23). The clinico-pathologic details for both OTSCC #1 and OTSCC #2 are summarized in the Supplementary excel file.

### Data pre-processing

Controlled-access single nucleotide variant files of the 425 whole exome sequenced cancers from the TCGA-HNSCC cohort analyzed in this study were obtained from Genomic Data Commons [GDC release v33.0 downloaded on 2022/05/11], for each variant caller used available (MuSE, MuTect2, VarScan2 and Pindel). Single nucleotide polymorphisms and reads that did not pass the variant caller’s filters were removed. Somatic mutation spectra of samples were generated using SigprofilerMatrixGenerator (v1.2) with its R wrapper, SigProfilerMatrixGeneratorR (v1.1).^18^ Somatic mutations included single base substitutions (SBS), small insertions and deletions (indels or IDs).

### Identification of rare mutational spectra

Signature bleeding may occur in the process of signature extraction when signatures identified in a small, highly mutated sample subset are ascribed to all samples. In order to detect mutation spectra outliers and mitigate potential signature bleeding, we first identified samples with high mutation burden by looking at mutation count distribution (Supplementary Fig. S1a). Next SBS96 mutation spectra of samples with more than 600 mutations were compared using cosine similarity. Hierarchical clustering of those samples (using 1-cosine as distance) showed presence of nine distinct mutation spectra (Supplementary Fig. S1b). Six of these spectra, with a mean spectrum exhibiting potential matches with COSMIC SBS signatures, were found in fewer than 5 samples and thus categorized as rare (Supplementary Fig. S1c). One spectrum, identified in two lip cancers sample and one OC cancer sample, exhibited similarities to the UV light signature (SBS7). Additional two spectra (present in three samples) resembled various combinations of SBS signatures of DNA mismatch repair deficiency (SBS15, SBS20 and SBS21). One spectrum (in one sample) manifested with a strong C > G pattern enrichment similar to the COSMIC SBS39 signature. Finally, one spectrum (in one sample) showed no resemblance to a COSMIC counterpart, and another (in one sample) was only observed as a result of MuTect2 variant calling and is thus a possible artifact.

### Extraction of de novo mutational signatures and sample clustering

After removing nine atypical samples with high mutation count (see above), signature extraction was conducted on the 416 remaining samples using SigProfilerExtractorR (default parameters; v1.1.4) for each variant caller (MuSE, MuTect2, VarScan2) separately. While up to 10 de novo signatures were extracted in both the SBS96 and SBS384 catalogs, the optimal number of mutational signatures was assessed based on both the mean sample cosine and average stability and set to 4 de novo signatures. Samples were finally clustered using K-means clustering algorithm into groups according to their signature relative contribution. Using the 384-channel signature dimension increased the stability across variant callers (Supplementary Fig. S2a), by reducing the number of patients switching between clusters, from 21% (88/416) to 16% (69/416). Consequently, we consistently used the 384 channel-based signature analysis throughout the study.

A heatmap of SBS384 signature extraction was generated for each variant caller (Supplementary Fig. S2b), and sample content in each cluster was compared among the variant caller-specific results, using a Venn diagram (Supplementary Fig. S2c). For each cluster, samples at the intersection of the 3 different variant callers were considered to be the most stable and were used for the final clustering. This final sample assignment to each cluster was used in the remaining analysis steps, involving 347 samples included in the subsequent analysis. These comprised 253 (73%) OCSCC and 94 (27%) LXSCC. The mean age of the patients was 62 (±12) years. The majority of patients were male (*n* = 246, 70%), 260 were smokers (75%), 92 (27%) had a documented history of alcohol consumption, and 18 (5%) were positive for HPV.

### COSMIC signature fitting

The all-COSMIC signature fitting by non-negative least squares (NNLS) was performed using MuTect2 variant caller-detected mutations, in samples with consistent clustering performance (see above). First, all COSMIC signatures were fitted to de novo extracted signatures using SigProfilerExtractorR (SPE) (v1.1.4), using default parameters. SPE assignment determined 8 COSMIC signatures: SBS1, SBS2, SBS4, SBS5, SBS6, SBS7a/b, SBS13, and SBS16 (Supplementary Fig. S3a), sample clustering based on the COSMIC signature decomposition and attribution did not reproduce the sample clustering based on the de novo signature contents (Supplementary Fig. S3b). This was mainly due to the activity broadly attributed to signature SBS5 (such as its 29.06% attribution in SBS384C) (Supplementary Fig. S3a). The SPE tool assumes that SBS1 and SBS5 are always present and active in all tissues.^55^ We hypothesized that this could overestimate the respective contributions of SBS1 and SBS5 in the analyzed data set.

To test this hypothesis, we performed a signature analysis complementary to the SPE decomposition by using the Mutational Signature Analysis (MSA)^83^ (v2.0) attribution approach. Using the signature set proposed by the SPE decomposition (SBS1, SBS2, SBS4, SBS5, SBS6, SBS7a/b, SBS13, and SBS16), we observed an overall reduced attribution of SBS5, resulting in restored stability of patient clusters (Supplementary Fig. S3c, S4d). Next, we applied the MSA-based attribution using the entire COSMIC signature set (version 3.2). As no SBS384 version of COSMIC signature was available at the time of the analysis, the SBS288 transcription strand bias versions of COSMIC signatures were used in each case, with an optimal threshold suggested by the MSA tool. Signature relative contribution was used to assess clustering stability. For final signature attribution, signature activities that included zero value inside the 95% confidence intervals were pruned to prevent overfitting. This approach resulted in cluster stability consistent with the de novo signature-based clustering (Supplementary Figs. S4). Thus, the overfitting of the SBS5 signature within the SPE decomposition step due to the default inclusion of the signature could be resolved by the MSA-based optimized refitting approach.

The possible masking of other signatures by SBS5 was based on several lines of evidence. Firstly, SBS5 in its transcriptional strand bias version is highly similar to signatures SBS16 and SBS92 (Supplementary Fig. S5). Indeed, MSA analysis could resolve the broad SPE-suggested presence of SBS5 into a biologically plausible assignment of a newly described tobacco-smoking-related signature SBS92 almost exclusively to the LX tumors in the SBS4-dominated tobacco-smoking cluster (Supplementary Fig. S4). Secondly, the resolution of SBS5 revealed an increased presence of SBS16 in smokers who were drinkers without compromising the stability of the de novo signature-based clusters (Supplementary Figs. S4).

To further characterize the behavior of SigProfiler fitting, we generated synthetic genomes. These simulated mutational profiles were designed to model a range of mutational scenarios by varying both mutational signature combinations and mutation burdens. For each synthetic sample, a fixed mutation count was assigned. Signature contributions were randomly drawn from a uniform distribution (0–1) and subsequently normalized to sum to one. The mutation probability for each mutation type was then computed as the weighted sum of the selected signatures, using these normalized contributions. Mutations were sampled with replacement according to these probabilities until the specified mutation count was reached. This approach identified signature over-attribution as a function of mutation count reduction (see Supplementary Fig. S6).

### Survival analysis

Overall survival (OS) and disease-free survival (DFS) were analyzed in the three clusters, mainly composed of oral cavity squamous cell carcinoma (OC-SCC) samples (SBS1, SBS1/APOBEC, and SBS16). The SBS4/SBS92 cluster, consisting almost entirely of laryngeal tumors, was excluded because these cancers differ in treatment and generally have a better prognosis, which could bias survival comparisons.

Overall survival (OS) was defined as the interval from diagnosis to death or last follow-up. Disease-free survival (DFS) was defined as the interval from diagnosis to the first documented tumor recurrence, death, or last follow-up. Patients without an event were censored at their last follow-up. Kaplan–Meier survival curves were generated for each cluster, and differences between survival distributions were assessed using the log-rank test, implemented with the survival (version 3.5-7) and survminer (version_0.5.1) R packages.

### Mutated genes under selective pressure identified by dN/dS analysis

Genes under selective pressure were identified by computing the non-synonymous to synonymous mutation (dN/dS) ratio using the dNdScv tool (v0.0.1.0) without covariate.^84^ A dedicated mutation set was created for this analysis using Pindel in addition to the other variant callers used (MuSE, MuTect2, VarScan2). Only SBS mutations or indels identified by at least two variant callers were retained. A mean VAF value was calculated for each resulting mutation. TCW (with W standing for A or T) was used as a template to identify APOBEC-like mutations, and XCG (with X standing for A, T, C, G) for SBS1-like mutations.

### Differential Gene expression and methylation analysis

For gene expression analysis, open-access RNA-Seq raw counts were downloaded from GDC [GDC release v33.0 downloaded on 2022/05/11]. The analysis was focused on primary tumors exhibiting consistent clustering performance, resulting in 342 samples (see Extraction of de novo mutational signatures and sample clustering section).

Normalization and differential gene expression analysis of NIRF-enriched clusters (SBS1 and SBS1/APOBEC) were carried out against the other OC cluster SBS16 using DESeq2 (version 1.38.3). As a control, the comparison of the SBS4/SBS92 tobacco smoking cluster to cluster SBS16 was performed.

Gene Ontology (GO) term enrichment analysis was conducted using the clusterProfiler package^85^ (version 4.6.2), on differentially expressed genes (DEG) with adjusted p-values less than 0.05 and a log fold change greater than 1.

Methylmix subtypes were derived from the study conducted by Brennan et al. ^62^. The NSD1 mutation status was determined using Ensembl VEP annotation extracted from single-nucleotide variant files. Only mutations with a high impact score were considered as inactivating mutations.

### Multiplexed Immunofluorescence

Multiplexed staining was performed on FFPE sections from two OTSCC cases (see Samples and clinical data above) using the following antibodies: anti-lipopolysaccharide (LPS) (mouse monoclonal, Invitrogen MA5-41632, 1:500, Cyanine2), anti-pan-cytokeratin antibody cocktail (mouse monoclonal, Invitrogen MA5-13203, 1:1 000, FITC), and anti-S100A7 (rabbit polyclonal, Invitrogen PA5-79947, 1:500, Cyanine5). Negative controls were performed using oral tongue cancer slides processed in parallel without the primary antibody. Staining was carried out on a Leica BOND RX automated slide stainer using the Bond Polymer Refine Detection Kit (Leica Biosystems, #DS9800), following the manufacturer’s instructions. Multiplex fluorescence imaging was performed with a Zeiss Colibri 7 scanner.

To analyze the fluorescent channel intensity in quantitative terms, regions of interest (ROIs) were delineated using the QuPath annotation tool (v0.50), and pixel-based measurements were obtained through the automated cell detection tool within each ROI. Mean fluorescence intensities were quantified for each marker, and the resulting data were visualized as pixel intensity-based maps (Supplementary Fig. S13).

### Cell-type deconvolution of RNA-seq data

Raw RNA-seq counts, initially annotated with Ensembl IDs, were converted to HUGO gene symbols using the *biomaRt* package (R/Bioconductor), retaining only one symbol per gene to avoid duplicates. Genes without a valid HUGO annotation were excluded from further analysis.

Immune cell composition was estimated using the *CIBERSORTx* online platform (https://cibersortx.stanford.edu), with the processed expression matrix as input. The LM22 leukocyte gene signature matrix was selected as the reference gene expression profile. To reduce cross-platform variation, batch correction was enabled using B-mode correction. All other parameters were kept at default settings.

### Statistical tests

Chi-squared (χ²) test was used to assess associations between categorical variables, including COSMIC signature activity by site and clinical data. The Wilcoxon rank-sum test was applied for comparisons of mutational process activity after COSMIC fitting and APOBEC3A/3B activity and expression levels.

## Supplementary information

Supplementary Table 1

Supplementary Figures

Supplementary file

## Data Availability

The data that support the findings of this study are openly available in the GDC Data Portal at https://portal.gdc.cancer.gov/. The code that supports the findings of this study is available from the corresponding authors upon reasonable request.
