# Supplementary material for: Mutational signature-based classification uncovers emerging oral cancer subtypes with distinct molecular patterns
Source: Int J Oral Sci. 2026 Apr 24;18:38. doi: 10.1038/s41368-026-00437-4 (PMC13109424; doi:10.1038/s41368-026-00437-4)
Supplement: Supplementary file 1 — Supplementary Table 1 [file 41368_2026_437_MOESM1_ESM.pdf]

		S			NS			unknown		
site	HPV	D	ND	unknown	D	ND	unknown	ND	unknown	Total
OC	-	72 (55)	19 (18)	111 (87)	8 (5)	18 (16)	52 (44)	0 (0)	6 (5)	286 (230)
	+	4 (4)	0 (0)	8 (5)	2 (2)	1 (1)	3 (3)	0 (0)	0 (0)	18 (15)
	unknown	2 (1)	1 (1)	3 (3)	0 (0)	2 (1)	1 (1)	1 (1)	0 (0)	10 (8)
LX	-	29 (22)	7 (6)	62 (54)	1 (1)	0 (0)	5 (4)	0 (0)	3 (3)	107 (90)
	+	2 (2)	0 (0)	1 (1)	0 (0)	0 (0)	0 (0)	0 (0)	0 (0)	3 (3)
	unknown	0 (0)	0 (0)	1 (1)	0 (0)	0 (0)	0 (0)	0 (0)	0 (0)	1 (1)
	subtotal	109 (84)	27 (25)	186 (151)	11 (8)	21 (18)	61 (52)	1 (1)	9 (8)	425 (347)

Initial dataset(Stable dataset)

Supplementary table 1 Distribution of samples based on the anatomical site and exposure status, and selection of a core subset characterized by a stable behavior during data preprocessing (see Methods).
