# Supplementary material for: Mutational signature-based classification uncovers emerging oral cancer subtypes with distinct molecular patterns
Source: Int J Oral Sci. 2026 Apr 24;18:38. doi: 10.1038/s41368-026-00437-4 (PMC13109424; doi:10.1038/s41368-026-00437-4)
Supplement: Supplementary file 2 — Supplementary Figures [file 41368_2026_437_MOESM2_ESM.pdf]

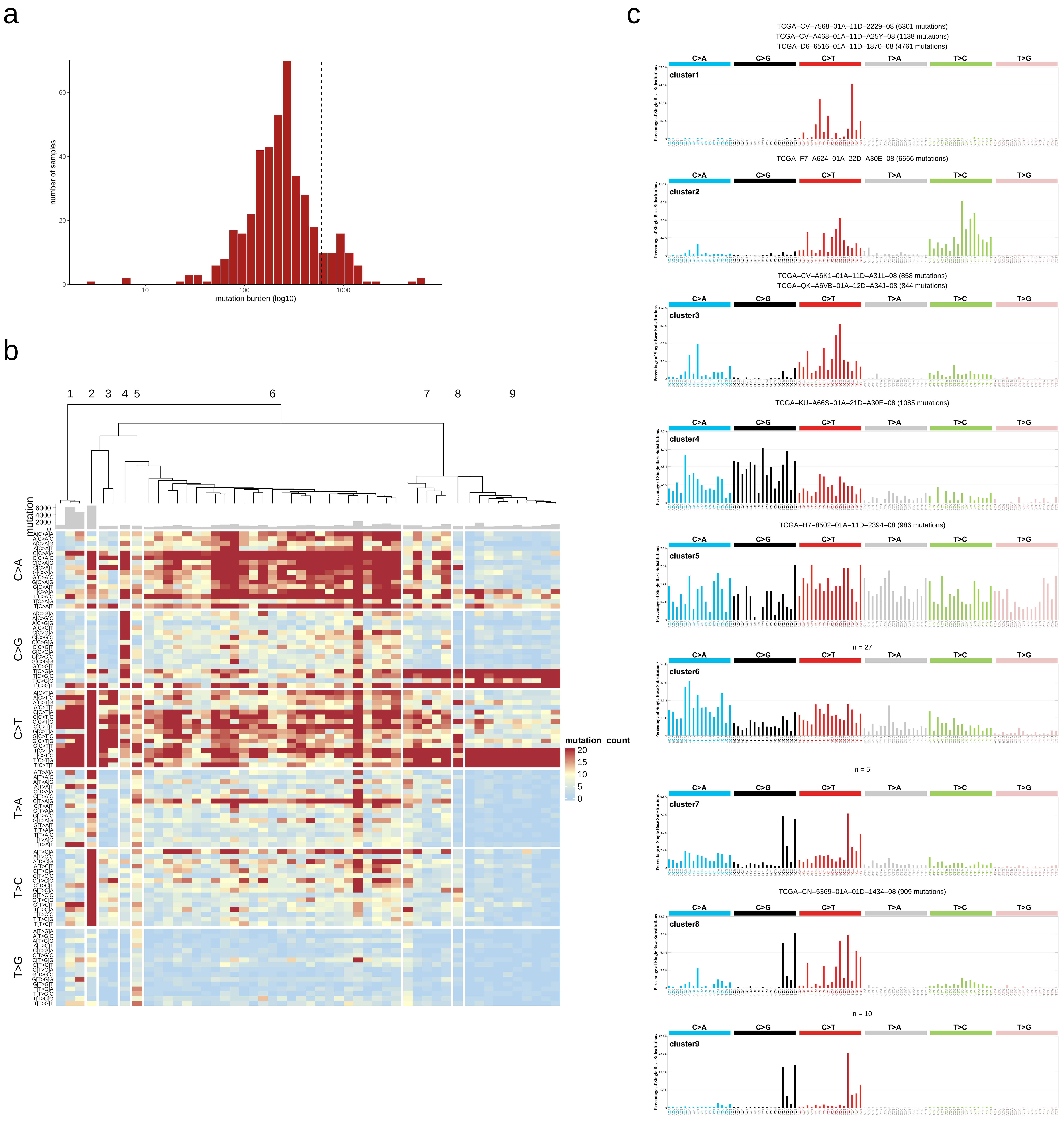

Supplementary figure S1 : Identification of rare mutational spectra. (a) Mutation burden distribution. (b) Heatmap representing mutation count in each SBS96 channel (row) for samples (column) with more than 600 mutations. Samples are ranked using hierarchical clustering with 1 - cosine as distance. Tree has been cut to create 9 clusters. (c) Mean mutation spectra for each cluster. For clusters with less than 5 samples, the sample names and mutation counts are indicated, otherwise only number of samples is given.

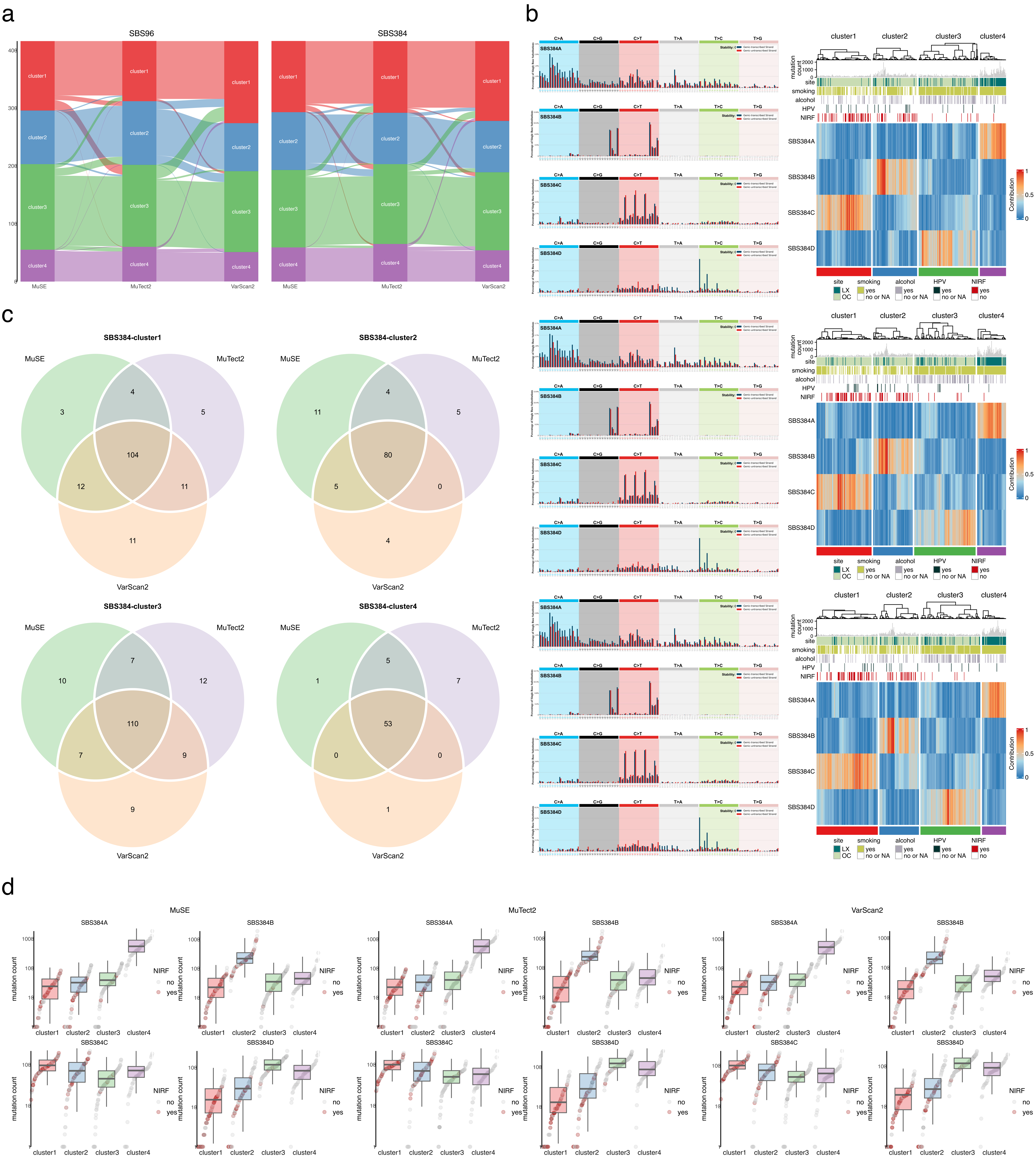

Supplementary figure S2: Cluster stability for different variant callers or mutation contexts. (a) Sample flow between clusters depending on the variant caller and the mutation context (SBS96 or SBS384). (b) Mutation spectra and sample clustering for Muse, MuTect2 and VarScan2 variant callers using the SBS384 context. (c) Venn diagram for MuTect2, VarScan2 and Muse variant callers in SBS384 context. For the rest of the study, clusters are composed of sample from the intersection of all variant callers. (d) Signature mutation burden for each cluster depending on variant caller.

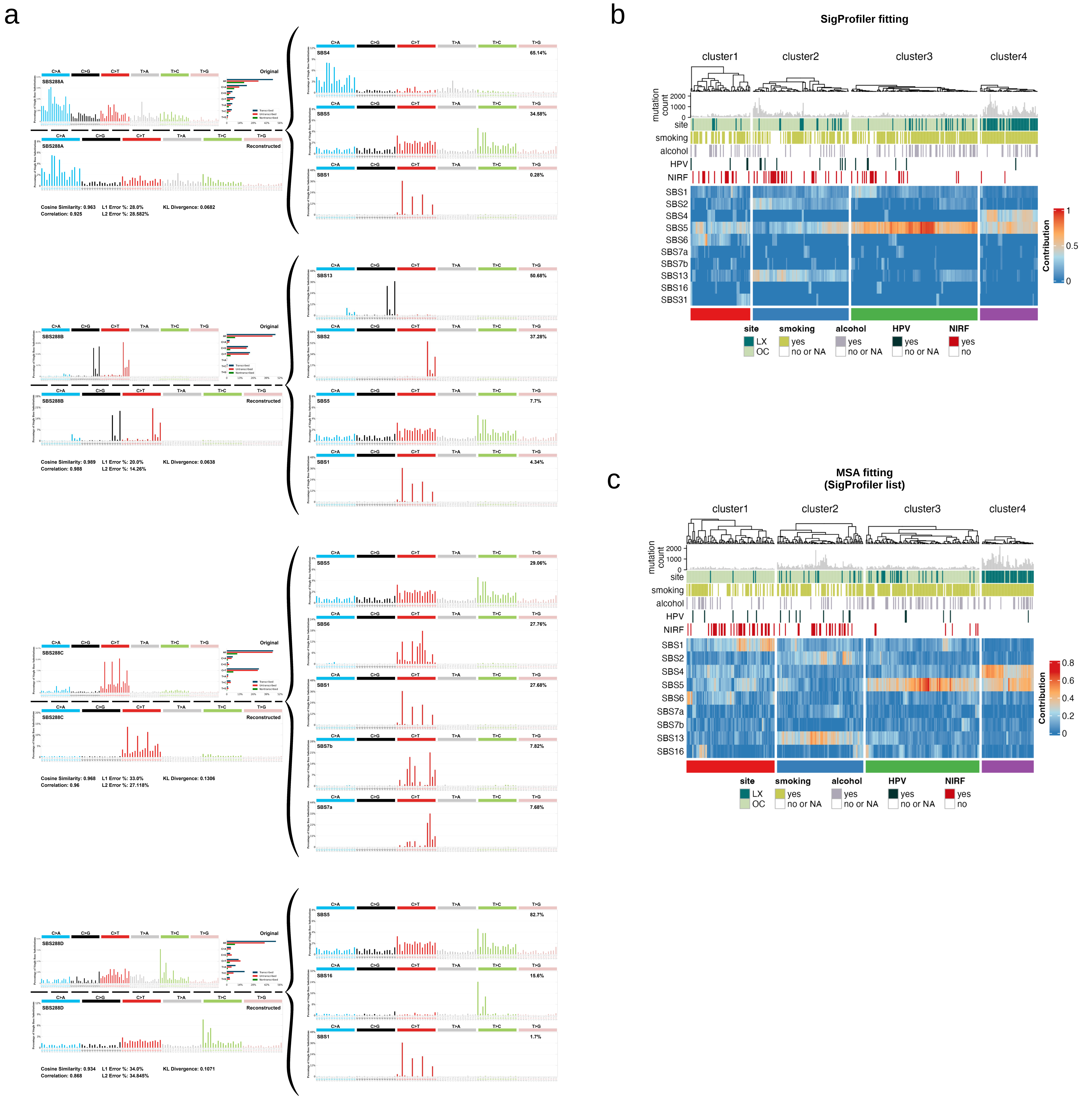

Supplementary figure S3 : SigProfiler fitting. (a) COSMIC decomposed solutions of de novo signatures by SigProfiler. (b) Heatmap and clustering according to SigProfiler signature attribution. (c) Heatmap and clustering according to MSA signature attribution using signature set proposed by SigProfiler fitting.

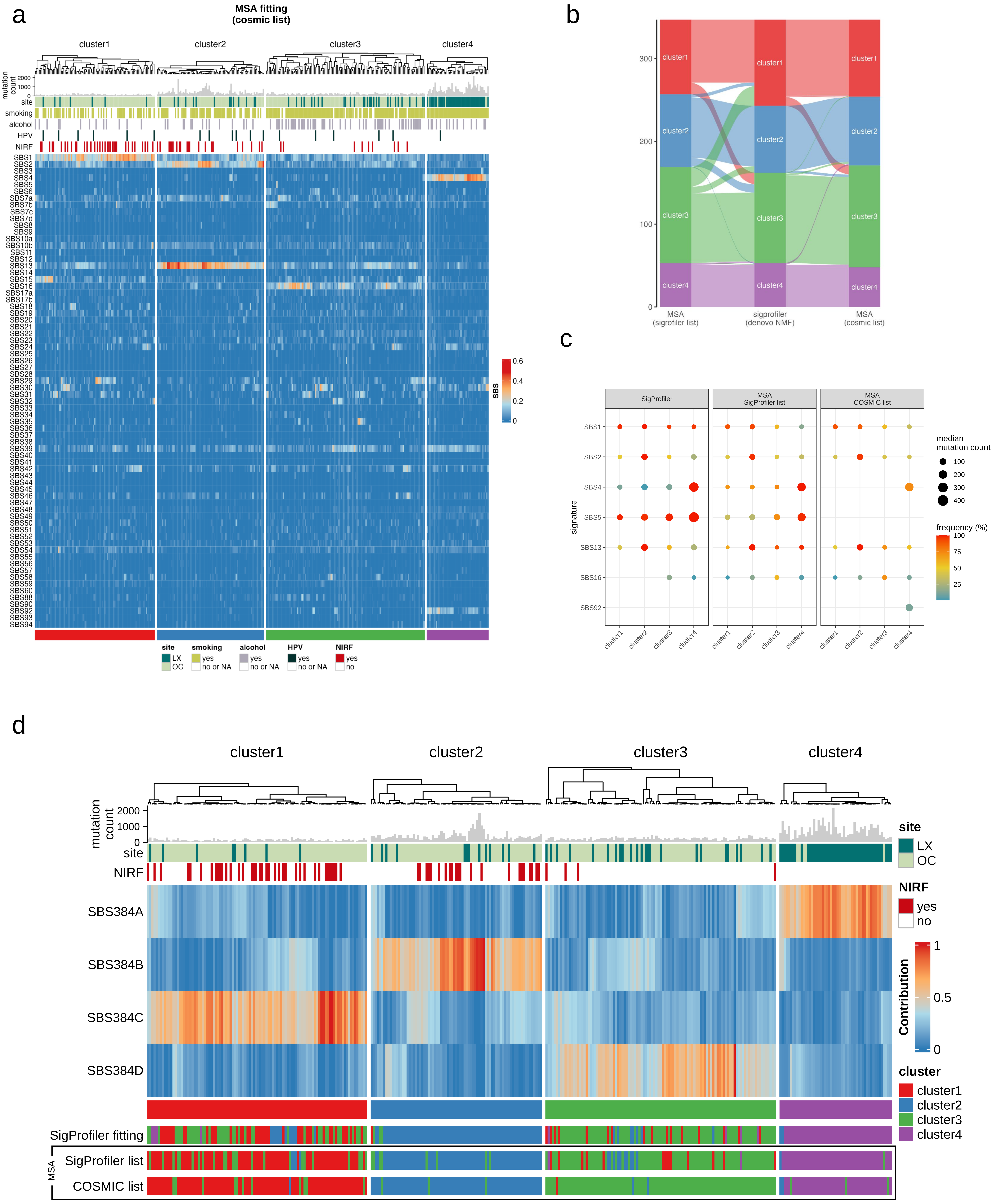

Supplementary figure S4: MSA vs SigProfiler attribution. (a) Heatmap and clustering according to MSA signature attribution using whole COSMIC as signature set. (b) Sample flow between clusters depending on the signature fitting strategy. (c) Bubble plot showing expected signature with median mutation count (size) and positive sample frequency (color) inside each cluster for the different fitting strategies. (d) Heatmap showing de novo mutational signatures across patients that restore the original clusters described in Fig. 1a. Heatmap tracks show the mutation count histogram, anatomical subsites (LX = larynx, OC = oral cavity), no identified risk factors (NIRF). Cluster color labels under the heatmap reveal the comparative sample redistribution outcomes after SigProfiler-based fitting and MSA fitting. The MSA-based fitting was performed by using the same signature set identified by SigProfiler fitting, or with a signature set encompassing all COSMIC signatures (v3.2).

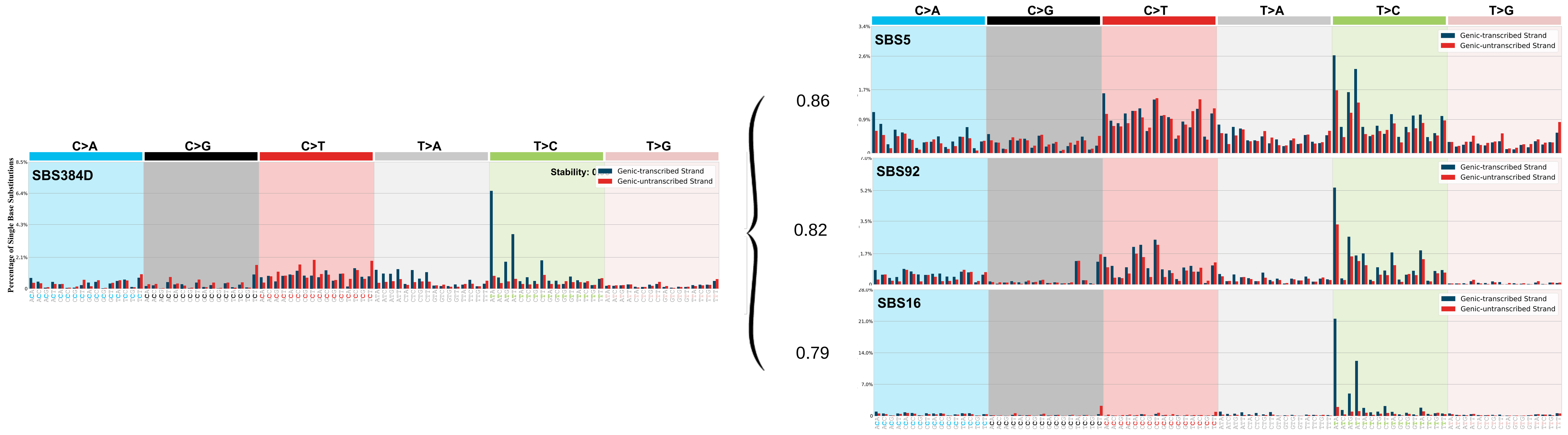

Supplementary figure S5 : De novo signatures identified after NMF extraction (A-D) with their putative COSMIC signature equivalent, with their cosine similarity shown.

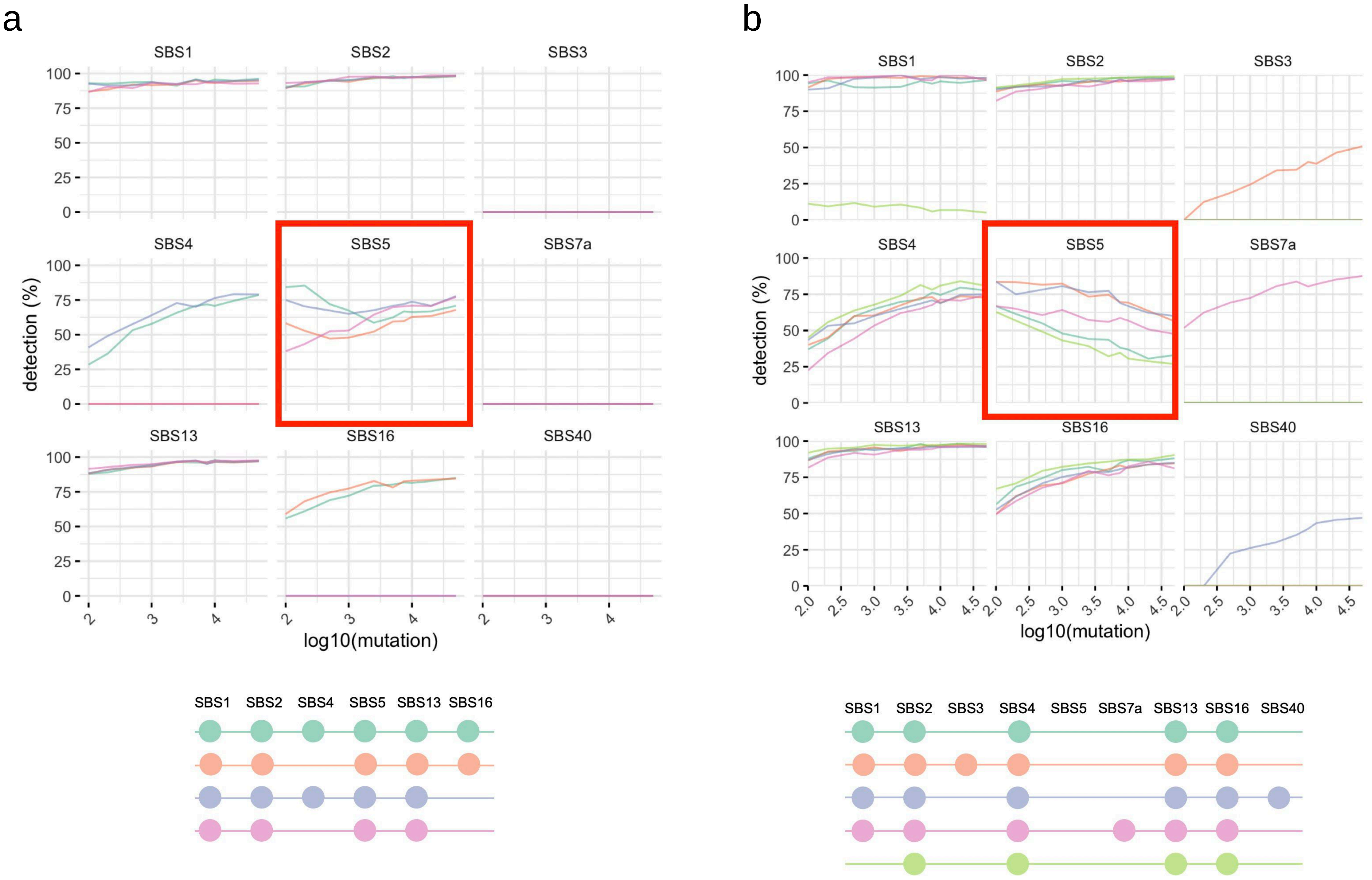

Supplementary figure S6: SBS5 detection using synthetic genomes across different signature combinations and mutation burdens. (a) scenarios including SBS5. (b) scenarios without SBS5. Each color represents a distinct combination of mutational signatures (indicated at the bottom). The presence of a given signature in the simulated sample is marked by a dot. At mutation burdens below 1,000 mutations, SBS5 detection (red square) was strongly influenced by the presence of SBS4 and SBS16. When SBS5 was absent, it was still consistently attributed across all combinations, with over-attribution decreasing as mutation burden increased.

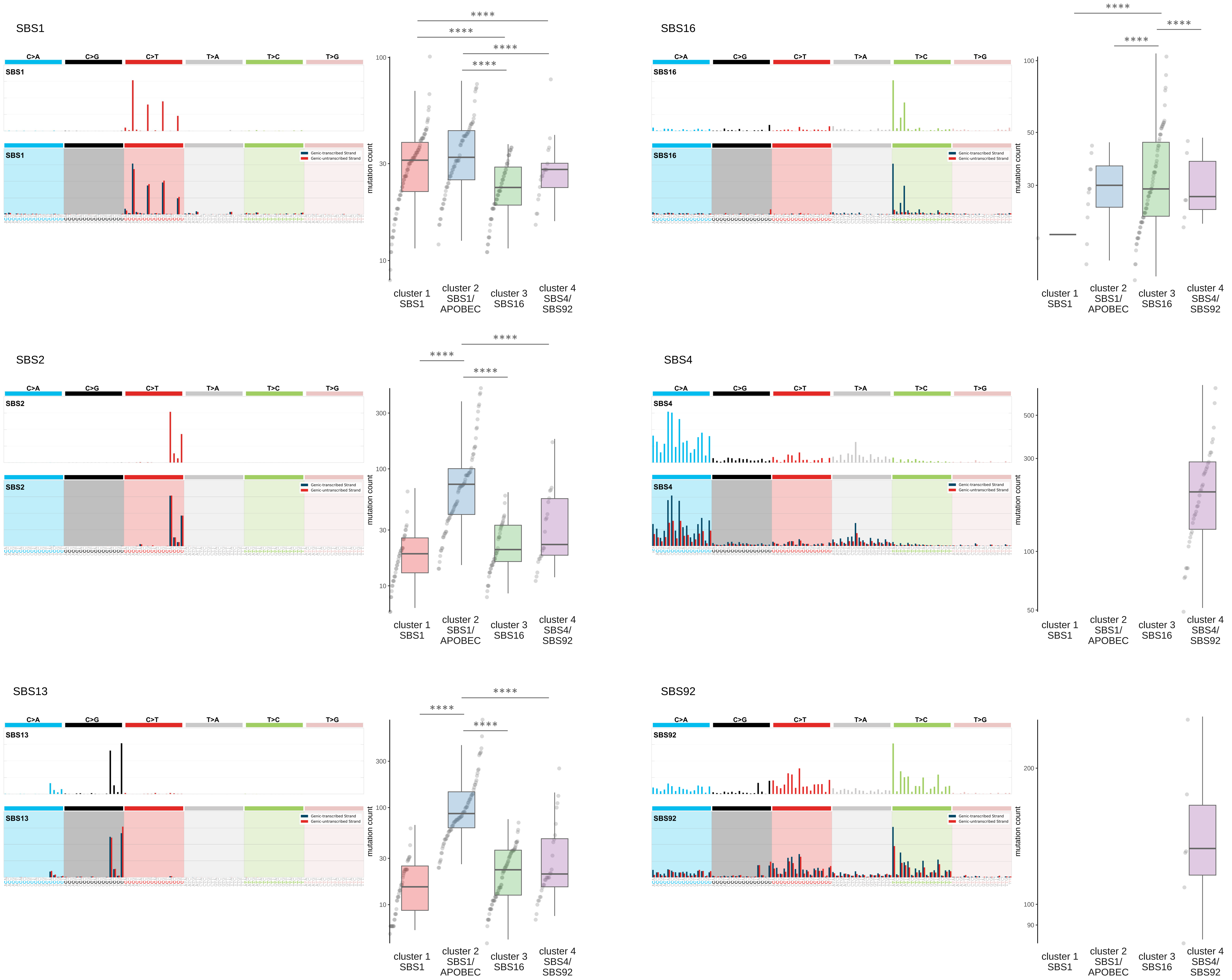

Supplementary figure S7: COSMIC signature fitting. Top-left panels: the 96-channel COSMIC signatures; bottom-left panels: the corresponding transcriptional-strand asymmetry signature versions. The histograms use the conventional COSMIC color coding for base substitution types. The boxplots (right panels) show the per-cluster distribution of mutation counts attributed to each COSMIC signature. Level of significance indicated by asterisks : * $p < 0.05$, ** $p < 0.01$, *** $p < 0.001$, **** $p < 0.0001$

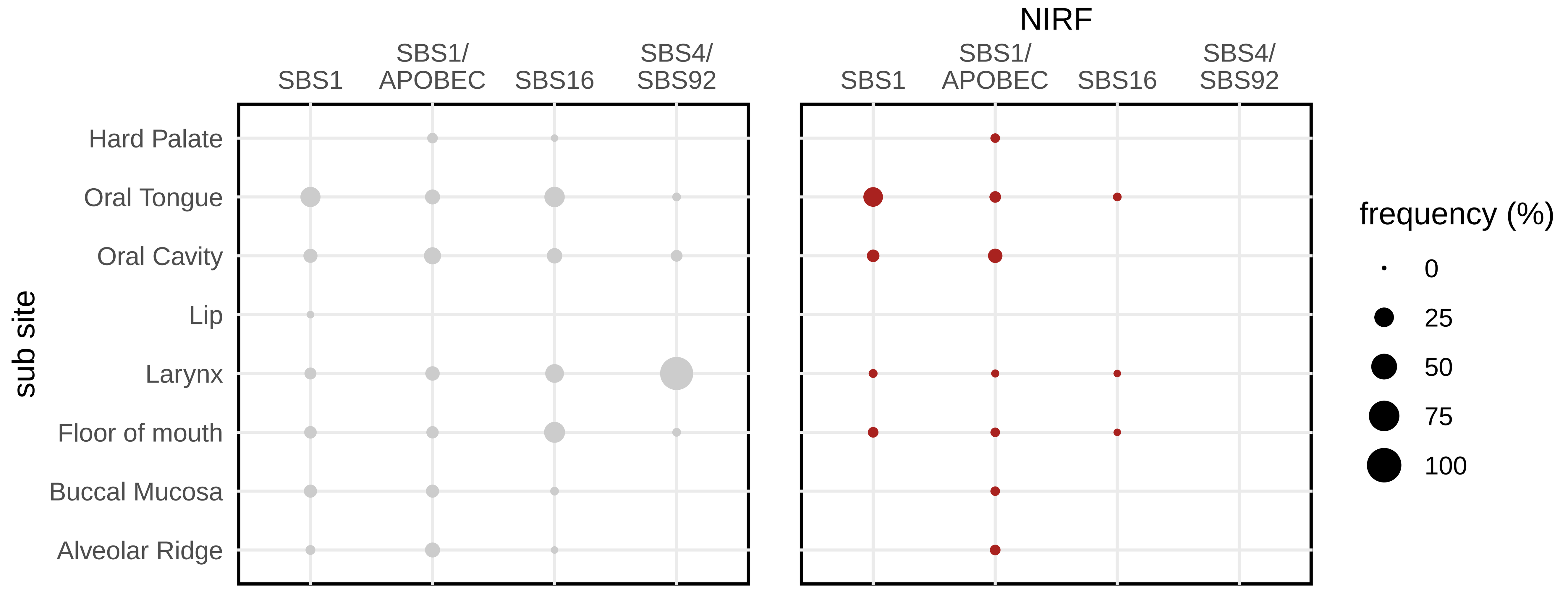

Supplementary figure S8: Bubble plot showing the anatomical sub-site distribution of the NIRF and non-NIRF patient subsets in each cluster

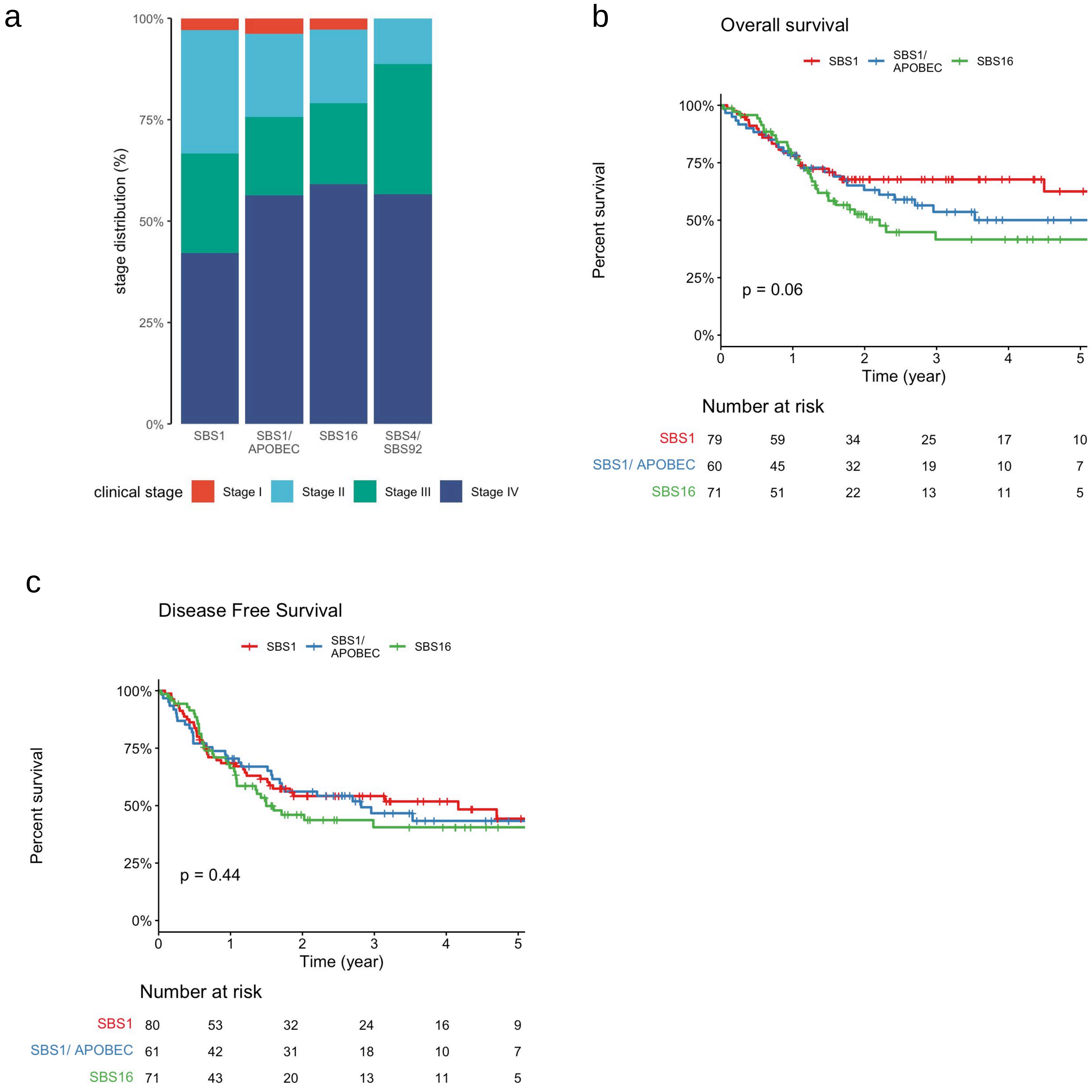

Supplementary figure S9 : Clinical stage and survival across clusters. (a) Distribution of clinical stages within each cluster. (b) Kaplan–Meier curves for overall survival by cluster, with the number of patients at risk displayed below. (c) Kaplan–Meier curves for disease-free survival by cluster, with the number of patients at risk displayed below.

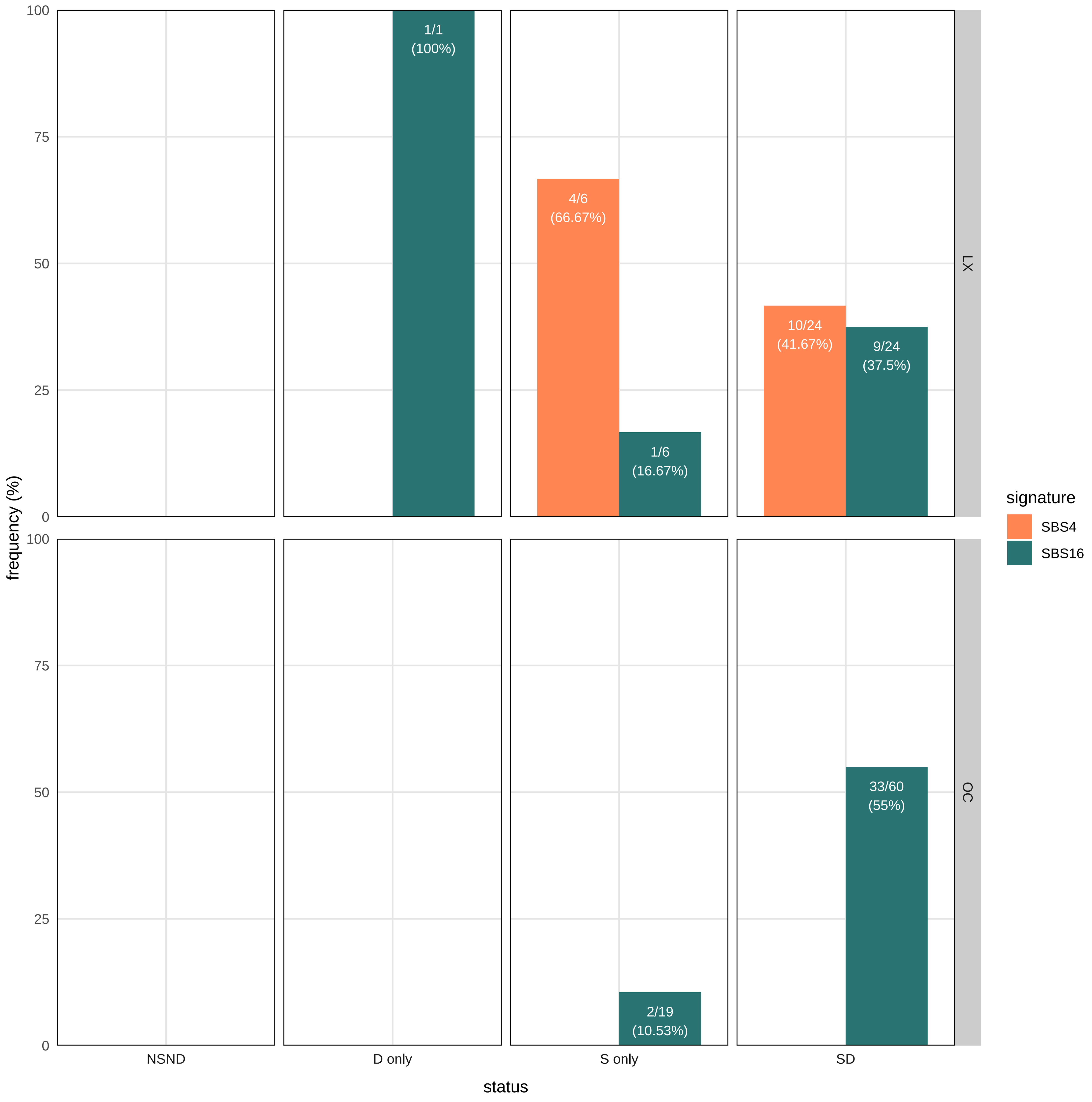

Supplementary figure S10: Relative distribution of samples positive for SBS4 and/or SBS16 according to site, smoking and alcohol consumption (non-smoker non-drinker (NSND), drinker only (D), smoker only (S), smoker and drinker (SD))

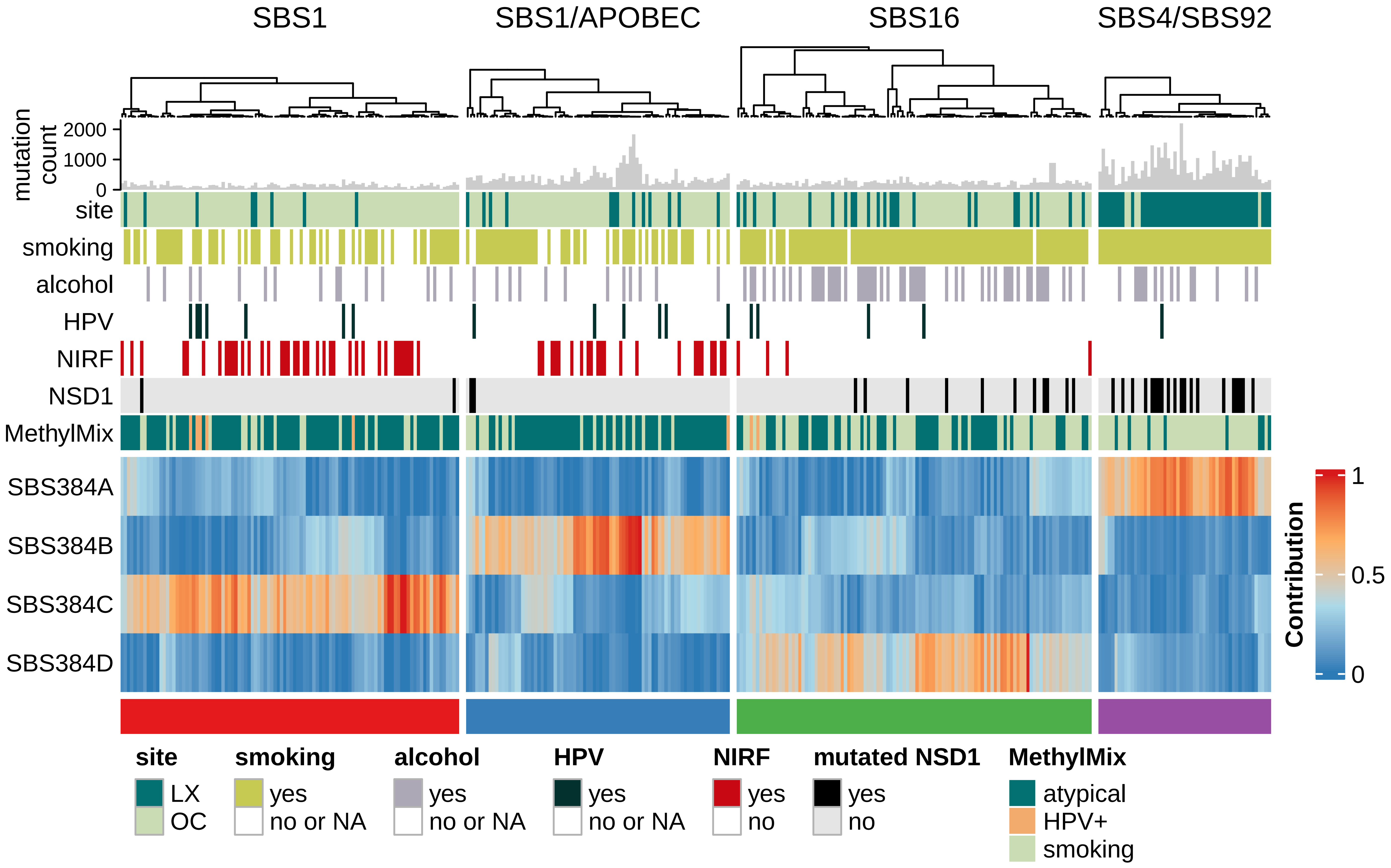

Supplementary figure S11 : Cluster methylation profile. Methylmix DNA methylation subtypes previously described by Brennan et al. 2017 (Non-CIMP/ CIMP Atypical, NSD1/ Stem-like Smoking and HPV+) have been added to cluster heatmap.

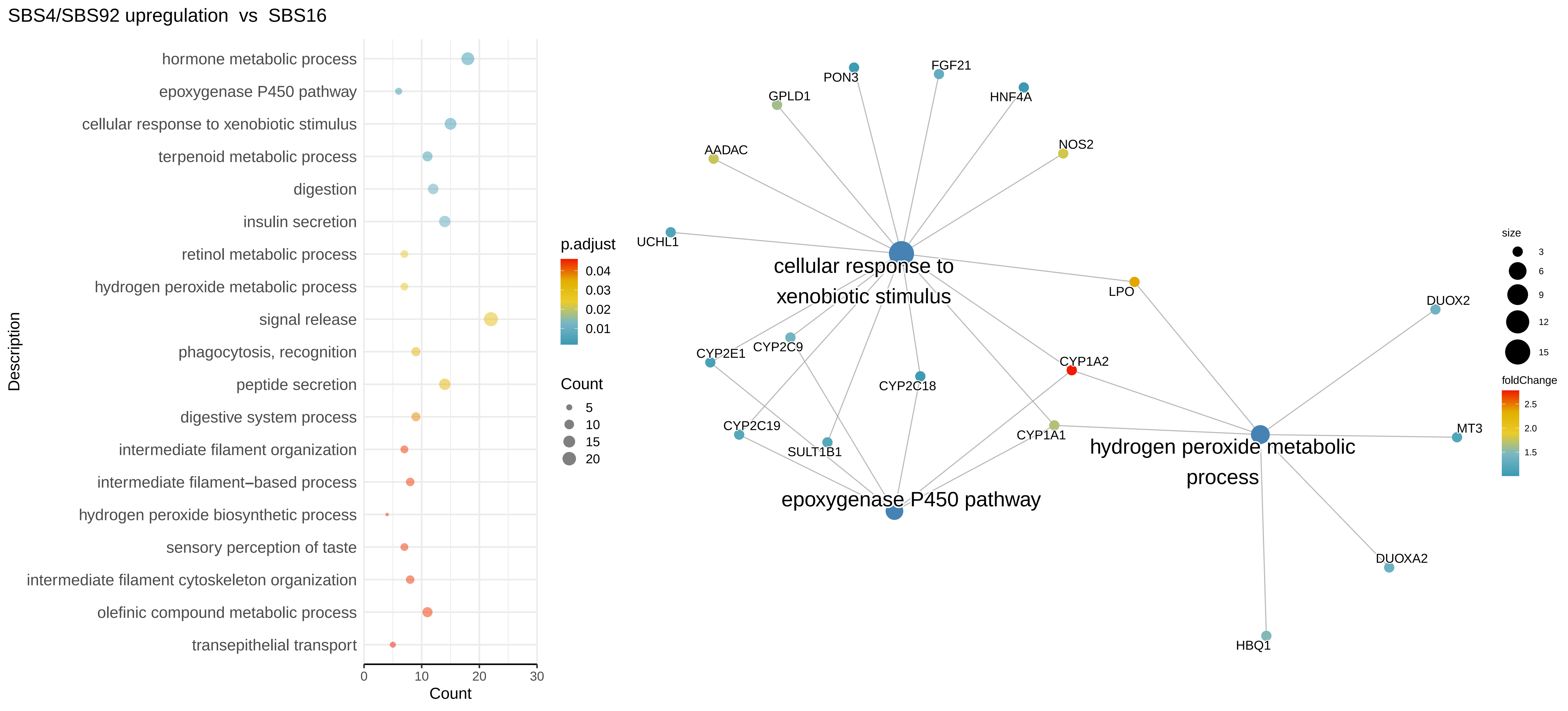

Supplementary figure S12: Tobacco cluster functional enrichment. Pathway enrichment for genes differentially expressed in tobacco smoking cluster SBS4/SBS92 against cluster SBS16. Pathways with an enrichment p-value < 0.05 are listed in left with count representing the number of genes enriched in the pathway. Network diagram (right) shows selected pathways and their respective upregulated genes.

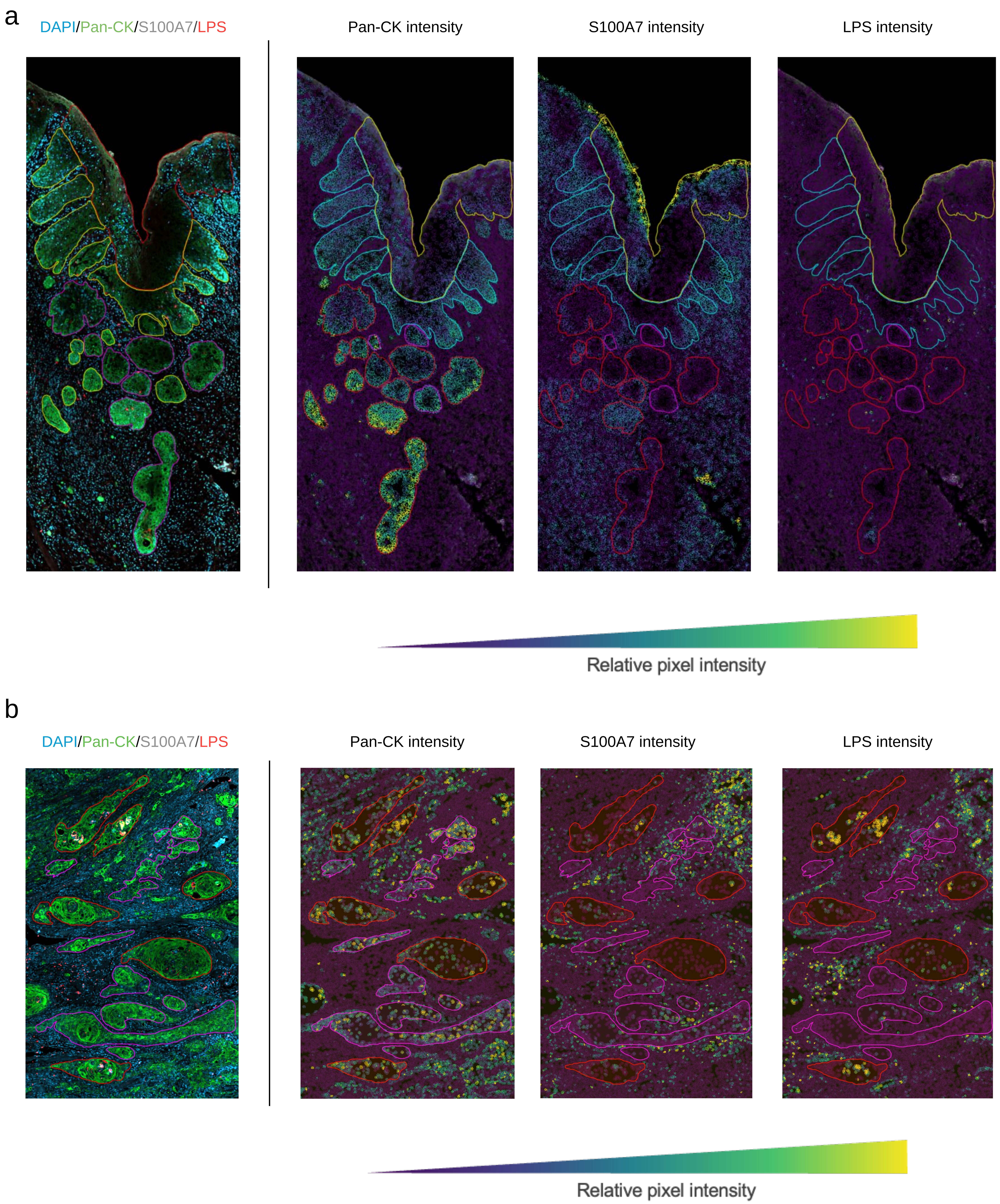

Supplementary figure S13: Spatial distribution of pancytokeratin (pan-CK), S100A7, and lipopolysaccharide (LPS) within oral tongue squamous cell carcinoma (OTSCC) tissues. The left panel depicts the intratumoral co-localization of the three markers in the analyzed sections (color-coded as shown above the panel), while the three right-hand panels display the corresponding pixel-intensity quantifications for each marker. (a) OTSCC specimen #1 (45-year-old patient, NIRF); (b) OTSCC specimen #2 (40-year-old patient, non-smoker, HPV-negative, light drinker).

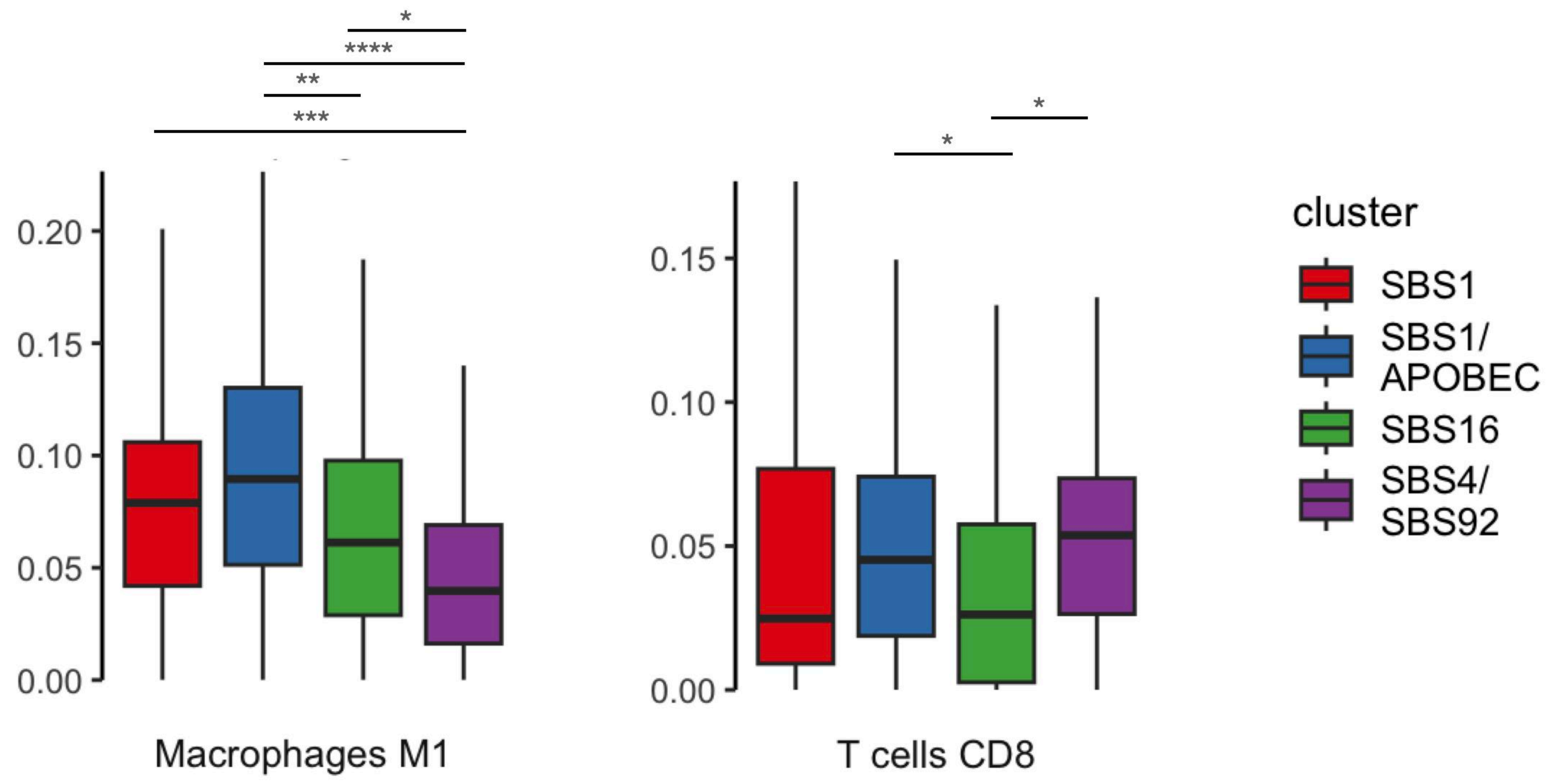

Supplementary figure S14: Estimated abundance of M1 macrophages and CD8$^+$ T cells across clusters, as inferred by CIBERSORT deconvolution. Significance indicated by asterisks : * p < 0.05, ** p < 0.01, *** p < 0.001, **** p < 0.0001.
